# Holistic pedestrian safety assessment for average males and females

**DOI:** 10.3389/fpubh.2023.1199949

**Published:** 2023-08-17

**Authors:** Christoph Leo, Anders Fredriksson, Ellen Grumert, Astrid Linder, Martin Schachner, Fredrik Tidborg, Corina Klug

**Affiliations:** ^1^Vehicle Safety Institute, Graz University of Technology, Graz, Austria; ^2^Volvo Car Corporation, Torslanda HABVS-VAK, Gothenburg, Sweden; ^3^Swedish National Road and Transport Research Institute, VTI, Gothenburg, Sweden; ^4^Mechanics and Maritime Science, Chalmers University, Gothenburg, Sweden

**Keywords:** active safety, HBM, holistic assessment, overall injury assessment, passive safety, pedestrian, sex-specific differences, VIVA+

## Abstract

**Objective:**

An integrated assessment framework that enables holistic safety evaluations addressing vulnerable road users (VRU) is introduced and applied in the current study. The developed method enables consideration of both active and passive safety measures and distributions of real-world crash scenario parameters.

**Methods:**

The likelihood of a specific virtual testing scenario occurring in real life has been derived from accident databases scaled to European level. Based on pre-crash simulations, it is determined how likely it is that scenarios could be avoided by a specific Autonomous Emergency Braking (AEB) system. For the unavoidable cases, probabilities for specific collision scenarios are determined, and the injury risk for these is determined, subsequently, from in-crash simulations with the VIVA+ Human Body Models combined with the created metamodel for an average male and female model. The integrated assessment framework was applied for the holistic assessment of car-related pedestrian protection using a generic car model to assess the safety benefits of a generic AEB system combined with current passive safety structures.

**Results:**

In total, 61,914 virtual testing scenarios have been derived from the different car-pedestrian cases based on real-world crash scenario parameters. Considering the occurrence probability of the virtual testing scenarios, by implementing an AEB, a total crash risk reduction of 81.70% was achieved based on pre-crash simulations. It was shown that 50 in-crash simulations per load case are sufficient to create a metamodel for injury prediction. For the in-crash simulations with the generic vehicle, it was also shown that the injury risk can be reduced by implementing an AEB, as compared to the baseline scenarios. Moreover, as seen in the unavoidable cases, the injury risk for the average male and female is the same for brain injuries and femoral shaft fractures. The average male has a higher risk of skull fractures and fractures of more than three ribs compared to the average female. The average female has a higher risk of proximal femoral fractures than the average male.

**Conclusions:**

A novel methodology was developed which allows for movement away from the exclusive use of standard-load case assessments, thus helping to bridge the gap between active and passive safety evaluations.

## 1. Introduction

In the year 2019, 48% of all road fatalities in Europe affecting the lives of VRUs amounted to 10,895 fatalities (1). Besides other strategies, car manufacturers and governments are expecting that the introduction and market penetration of new active safety systems, such as AEB and emergency evasion, respectively, will significantly change the overall number of pedestrian accidents (2–9). However, the studies also conclude that it will not be possible to avoid all VRU accidents.

Pedestrian AEB Systems are already being assessed in current European New Car Assessment Program assessments (10) and have, therefore, gained increasing importance in the last few years. From 2024 onwards, they will become mandatory (11). Hence, the distribution of crash configurations of VRU accidents is predicted to change in the near future. Consequently, new priorities of crash configurations will have to be considered in crash performance testing since simply analyzing contemporary accidents will not be sufficient. Hence, it is also necessary to predict how priorities will change due to the continued implementation of crash avoiding safety systems, such as different versions of AEB. In case of unavoidable accidents, the remaining impact configuration will be significantly affected, due to active safety systems influencing the relative velocity between VRUs and vehicles (12, 13). Therefore, developing a holistic assessment procedure, considering the ratio of avoided cases as well as the effect on unavoidable cases, is essential.

Regarding virtual pre-crash simulation, the effectiveness of active safety systems (such as AEB) can be assessed on a variety of generic or real-world conflict scenarios (6, 14–19). A common approach to determining testing scenarios involves using reconstructed real-world accidents. Each conflict scenario is represented in a detailed, explicit way, comprising trajectories of all accident participants based on evidence collected in case records. The use of reconstructed accident scenarios is a fundamental method for the evaluation of safety systems. However, they also represent the extremes of possible scenarios, which are determined and influenced by many different parameters that may not be present in a sampled accident. An attempt to tackle this issue is applying the stochastic determination of conflict situations (20). Such an approach attempts to determine potential conflict scenarios by objectively analyzing the variation of “possible” influencing factors. However, this leads to a large number of scenarios to be considered, where the question remains how to interpret the result in terms of the achieved benefit from the system for non-avoided crashes.

For the assessment of the injury risk for VRUs in the remaining cases, different options could be applied:

- Regression models have been developed to predict the injury severity as a function of collision speed (15, 17, 21–24). As also the age and not only the collision speed influences the injury Niebuhr et al. (25) and Wisch et al. (26) have developed speed-dependent injury risk curves for different age groups. This, however, does not allow for consideration of the specific passive safety measures of a specific car that shall be assessed.
- Hardware tests with impactors which are used in current consumer information (27) and regulatory tests (28) have the limitation that only limited injury types (29, 30) can be assessed and they have been designed for a limited speed range only (31). Furthermore, legform impactors have only been designed to represent the average male stature. This is a shortcoming, as studies on real-world data have shown significant differences in injury patterns between males and females (32, 33). For example, it was found that the odds of sustaining skeletal injuries to the lower extremities (incl. pelvis) are significantly higher for females (32). Furthermore, hardware tests come along with a limited number of affordable test conditions. Previous studies aiming for a holistic assessment have therefore used regression models (34) to interpolate in between impact speeds with the limitation that the angles and head impact speeds depend highly on the collision scenario and are hard to predict (13).
- Crash test dummies (35–37) representing the pedestrian are hardly used in research. In contrast to the impactors, more body regions can be assessed and the interaction of the entire body resulting in different impact conditions of the body parts considered. However, the dummies are currently only available in the stature of an average male and have only been so far tested in a specific speed range.
- Human Body Models (HBMs) can be used for the virtual assessment of the injury risk for VRUs in accidents. As HBMs are more biofidelic than traditional crash test dummies, it is possible to gain more in-depth knowledge regarding kinematics and sustained injuries. Moreover, it is possible to develop a specific injury risk curve for different body regions and injury types (38). This helps to evaluate the passive safety of the car in a much more detailed way than just using the methods mentioned above. One of the benefits of using HBMs in a virtual testing environment is that a baseline HBM can easily be morphed into many anthropometries (39, 40). However, previous studies (13) have mainly used the classic anthropometries (5th percentile female, 50th percentile male and 95th percentile male). To overcome this, and to be able to evaluate males and females in an equal way by using HBMs, the VIVA+ (41) models can be used. These VIVA+ models are available as average female (50F) and average male (50 M).

The aim of this study is to develop and test a holistic assessment method that bridges the gap between active and passive safety assessments considering variability observed in field data and considers females and males equally. We aimed to combine stochastic evaluations of an active safety system with injury risk predictions based on HBMs for the evaluation of the passive system in the remaining crashes. For the sex-specific assessment of the in-crash performance and assessment of passive safety systems, the VIVA+ models (50M and 50F) have been used in this study. Finally, we aimed to investigate the sex-specific differences in protection for an exemplary safety system by applying the developed methods.

## 2. Materials and methods

**Figure 1:**
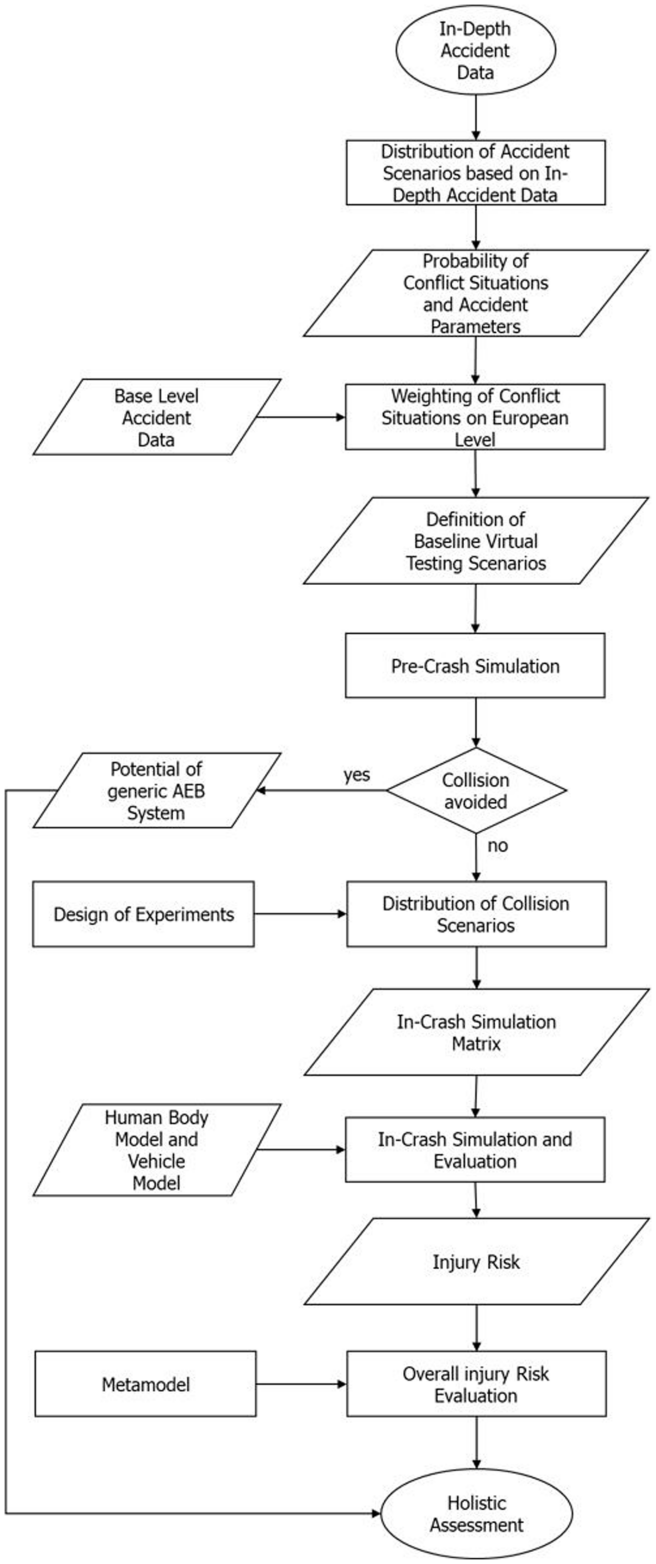
Holistic assessment workflow.

The integrated assessment framework which enables holistic safety evaluations as illustrated in Figure 1 consists of the following main steps which are described in more detail in the related sections.

*Step1:* In-Depth Accident Data analyses – Probability of Accident Parameters (2.1): Analyses of motion sequence parameters (e.g., initial velocity) and calculation of the probability for a given accident scenario which can then be used to define the virtual testing scenarios of pre-crash simulations. The related probabilities for each conflict situation used in this study can be found in the Supplementary material of this study.

*Step 2:* Base Level Accident Data analyses – Weighting of Conflict Situations on European Level (2.2): Analyses of European accident data according to injury severity (slight, severe and fatal) and corresponding conflict situations to weight the virtual testing scenarios for pre-crash simulations on European level. The calculated weighting factors for different injury severities and conflict situations can be found in the Supplementary material and on the OpenVT platform.^1^

*Step 3:* Probability of a specific virtual testing scenario for pre-crash simulations (2.3): Based on the probabilities obtained from the accident data calculated in *Step 1* and *Step 2*, the virtual testing scenarios for the pre-crash simulations can be defined. A related occurrence probability can be calculated based on real-world accident data for each of the defined virtual testing scenarios. This leads to a catalog of virtual testing scenarios which represents the baseline. The used pre-crash tool for this study as well as the defined virtual testing scenarios based on the results of *Step 1* and *Step 2* is also available on the OpenVT platform.^2^

*Step 4:* Pre-Crash Simulation (2.4): To show the potential of an active safety system (AEB) pre-crash simulations can be conducted with the help of the defined catalog of virtual testing scenarios in *Step 3*. The potential of the AEB can be calculated by summing up the avoided virtual testing scenarios with its related occurrence probability. The unavoidable virtual testing scenarios can be clustered by their parameters (e.g., collision velocity). Together with the occurrence probability of a specific unavoidable virtual testing scenario, a collision scenario can be defined. These defined collision scenarios can then be used to investigate the passive safety performance of the car with the help of HBM in-crash simulations. The results of the pre-crash simulations can be found in the results section of this study.

*Step 5:* In-Crash Simulation (2.5): From the catalog of collision scenarios defined in *Step 4*, a reasonable number was selected by an DoE to be considered for in-crash simulations between the HBM and the vehicle. The script used to design the simulation matrix from a catalog of collision scenarios is available on the OpenVT platform.^3^

*Step 6:* Injury Risk Evaluation (2.6): The in-crash simulations performed in *Step 5* have been evaluated according to the injury risk for different body regions. With the help of a metamodel, the injury risk for the other collision scenario defined in *Step 4* and not considered in the in-crash simulations can also be predicted to get the overall injury risk. The scripts for analyzing the in-crash simulations from VIVA+ simulations can be found on the OpenVT platform (see footnote 3).

### 2.1. In-depth accident data analyses – probability of accident parameters

To derive accident scenarios that can currently be observed in road traffic, data from three European, in-depth accident databases was investigated. The German In-Depth Accident Study (GIDAS) (42), the Austrian Central Database for In-Depth Accident Study (CEDATU) (43, 44) and the Volvo Cars Pedestrian Accident Database (V_PAD) (45) were used for this study. To describe accidents between pedestrians and passenger cars, the parameters given in Supplementary Table S1 from the accident databases were collected and analyzed. Moreover, the data was analyzed according to conflict situations. A conflict situation roughly describes the moving pattern of the participants (e.g., pedestrian is crossing from the left and the vehicle is driving straight). To describe the conflict situations, the proposed method by Lindman et al. (45) was used. All accident parameters were collected according to the different conflict situations. Graphics of all the used conflict situations can be found in the Supplementary Figure S1. Since the base level database CARE was also examined for further analyses, these conflict situations had to be clustered, as such a detailed description is not available in these base level databases. This results in the following clustered conflict situations: Car straight on – Pedestrian crosses, Car turns – Pedestrian same direction (SD), Car turns – Pedestrian oncoming, Car and Pedestrian in longitudinal traffic. Which conflict situation belongs to which cluster can be seen in Supplementary Figure S2. Supplementary Table S2 lists the available number of accidents regarding investigations for the different databases, injury severities and conflict situations.

**Figure 2:**
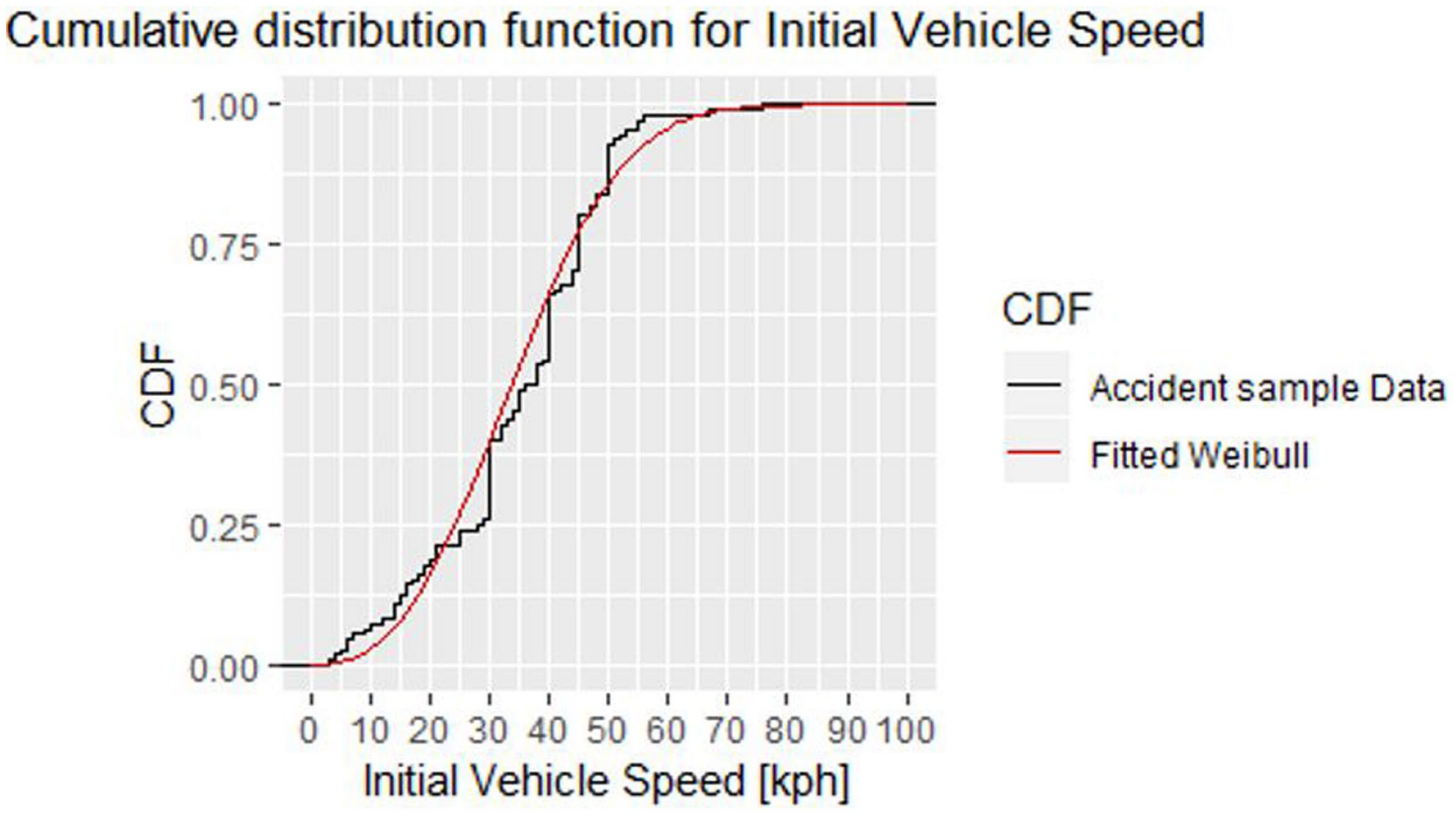
Fitted Weibull distribution on the accident data from V_PAD, GIDAS, and CEDATU for initial vehicle speed ending in a slightly injured pedestrian accident for SCPPL.

For the probability analysis of the motion sequence parameters, and particularly the initial velocities of the vehicle and the VRU, the data of GIDAS, V_PAD and CEDATU have been summarized for each conflict situation. As a next step, after the probability analysis, a Weibull distribution was fitted for each conflict situation and injury severity. To fit the Weibull distributions to the accident data, the software package R (46, 47) was used. An example of the final Cumulative Distribution Function (CDF) for the initial vehicle velocity in pedestrian accidents (resulting in slight injuries) for scenario SCPPL (straight crossing path, pedestrian from left) is shown in Figure 2.

It is possible to fit the Weibull distribution to a given data sample by determining the scale and shape factor. As two parameters are required to fit the distribution, only conflict situations are used where more than two data samples (accidents) are available. The defined Weibull distributions can then be used to determine the likelihood of a certain initial speed per conflict situation. This likelihood is needed to calculate the probability of a specific virtual testing scenario occurring in real life. How this is done is described in Section 2.3.

### 2.2. Base level accident data analyses – weighting of conflict situations on European level

As seen in Supplementary Table S2, in-depth accident data is only available for a relatively small number of cases, and the data does not represent European statistics, only national level statistics. To weight the probability of the different conflict situations on European level, the base level accident database CARE (48) was included in this study. CARE is a European database that includes all national police reported road statistics for 26 European countries (48). The data from the CARE database was categorized according to the clustered conflict situations. However, as mentioned above for the base level accident databases, no detailed description of the conflict situations is available. In addition, no information regarding the motion sequence parameters (e.g., initial velocities of the vehicle and the VRU) is available; the data can only be collected according to the injury severity of the pedestrian. Therefore, the in-depth databases V_PAD, GIDAS and CEDATU were used to allow for distribution of accidents into a specific conflict situation—a necessity when estimating the probabilities of each conflict situation. V_PAD, GIDAS and CEDATU represent the baseline samples, having assumed that they provide probabilities representing Europe within one cluster and severity. Databases with few observations do not contribute to the result as much as databases with a large sample of incident statistics. GIDAS holds the highest number of reported minor accidents (*n* = 354), whereas CEDATU holds the highest number of reported fatal accidents (*n* = 153). For severe accidents, the number of reported cases was similar among the databases. Furthermore, it can be generally concluded that there are only a few observable cases of severe and fatal accidents resulting in uncertain estimations and results that are sensitive to the cluster division. The estimated and observed proportion of pedestrian accidents were similar, which indicates that independent of the database, the distribution of accidents is similar for conflict situations within a cluster. To obtain the overall probability of each cluster situation, the probabilities within each cluster are multiplied by the probability of an accident belonging to a specific cluster, based on the division of accidents clusters within CARE. The clusters Unspecified and Others are excluded due to the large number of observations in these categories and their resulting impact on the probabilities. The hypothesis is that accidents categorized as Unspecified should belong to one of the other clusters with the same distribution as the ones already divided into the other clusters, although, it cannot be confirmed. Supplementary Table S5 shows the derived probabilities for each injury severity, and Supplementary Table S6 shows the derived probabilities for each type of conflict situation for the different injury severities. The final probabilities are, in most cases, comparable to the databases.

**Figure 3:**
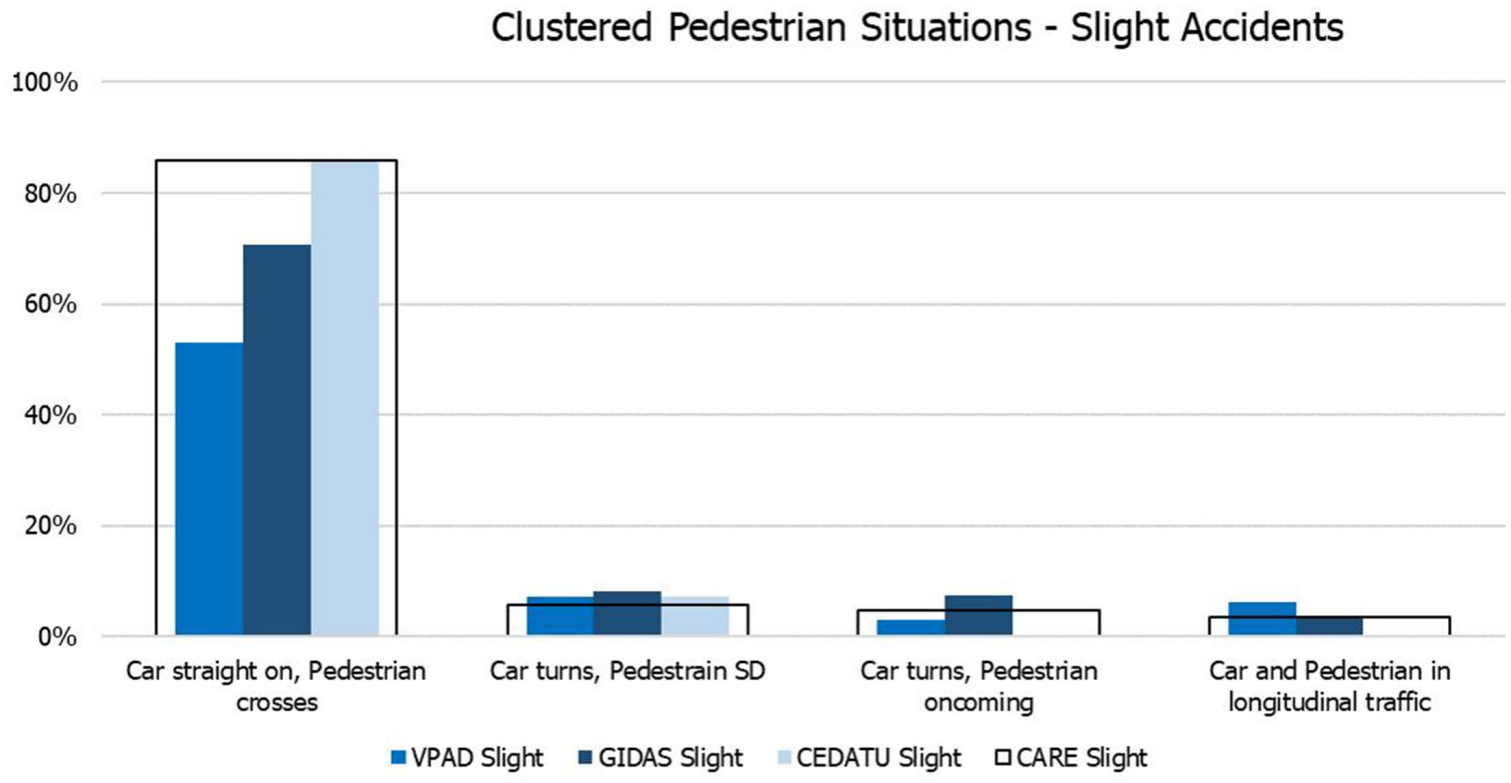
Probability for clustered pedestrian conflict situations based on slight accidents.

To derive the probabilities of injury severity and the probability of a specific conflict situation in each cluster of conflict situations on European level, the Iterative Proportional Fitting (IPF) method is used. For a detailed description of the IPF method, the mathematical procedure and its corresponding properties, see Fienberg (49), Plackett et al. (50) and Norman (51). IPF is only useful when the accidents are distributed similarly among the different conflict situations for the included in-depth databases, which is assumed to be true when comparing the distribution of conflict situations within a specific cluster. However, since the distribution of accidents between clusters differs for the included databases, the final probabilities are calculated based on the cluster distribution of accidents given by CARE. Only the cluster specific conflict probabilities are calculated with the IPF. Furthermore, the probabilities within each cluster are calculated per injury severity due to the fact that some of the databases include just one category of injury severity. In Figure 3, the result from the CARE based IPF is compared with the in-depth accident data V_PAD, GIDAS and CEDATU for accidents resulting in slight injuries.

### 2.3. Probability of a specific virtual testing scenario for pre-crash simulations

Next, the occurrence probability of a virtual testing scenario P(scenario) , shown in Equation 1, was calculated by multiplying the individual occurrence probabilities of the boundary conditions. It was decided to only differentiate between the different conflict situations (CS) and injury severities (IS), which are based on European level, while the probabilities for the initial vehicle velocity ( $v_{veh}$ ), initial pedestrian velocity ( $v_{VRU}$ ) and road conditions (RC), which are based on national level (in-depth data), were treated as statistically independent. On the one hand, this decision was based on the size of the data sample, where a statistically dependent approach would have led to too precise a subdivision, and therefore, too many categories with missing data. On the other hand, the data is a random sample of real-world accidents, where any parameter combination can occur.


(1) $$\begin{array}{l} P\left(scenario\right)=P\left(IS\right)*P\left(CS|IS\right)*P\left(v_{veh}|IS,CS\right)*\\ \;\;\;\;\;\;\;\;\;\;\;\;\;\;\;\;\;\;\;\;\;\;\;\;\;\;\;\;\;\;\;\;\;\;\;P\left(v_{VRU}|IS,CS\right)*P\left(RC|IS,CS\right) \end{array}$$

The individual probabilities in Equation 1 were derived from the analyzed, in-depth and base level accident data. The analyzed data was split into three different IS categories (minor, severe, fatal). For each IS, there is a certain probability of their occurrence P(IS) , which is shown in Supplementary Table S5. Within these IS categories, the occurrence probabilities for a certain CS P(CS|IS) are also known and shown in Supplementary Table S6. The CDF of the fitted Weibull distributions for the initial velocities ( $F_{v_{veh}},\;\;F_{v_{VRU}}$ ) were equally split into five percentile steps for $F_{v_{veh}}$ and ten percentile steps for $F_{v_{VRU}}$ . The resulting speeds were utilized as initial speeds in the pre-crash simulation. This approach also led to an equally distributed occurrence probability for the initial speeds $P\left(v_{veh}\mathrm{|,}IS\mathrm{|,}CS\right)$ and $P\left(v_{VRU}\mathrm{|,}IS\mathrm{|,}CS\right)$ .

The last parameter taken into account, in order to describe a testing scenario, was the road condition (RC). Since different databases use different classification systems for RC, it was decided to use the category “non-dry” for all categories not belonging to dry road conditions. This categorization led to the probabilities for dry and non-dry road conditions, given a certain injury severity, shown in Supplementary Table S7.

### 2.4. Pre-crash simulation

With the approach described in Schachner et al. (52), an individual virtual testing scenario can be built by providing the conflict situation, initial speed of the vehicle $v_{Veh}$ , initial speed of the VRU $v_{VRU}$ , road condition (dry/non-dry) and collision point (point where vehicle and VRU trajectory cross each other). For each virtual testing scenario of the catalog, a unique Identifier (ID) is created by combining all the scenario parameters. Overall, the derived catalog consists of more than 61,000 baseline scenarios for the pedestrian.

For each virtual testing scenario, a baseline simulation (no driver reaction or AEB system intervention) was performed as well as a simulation with a virtual AEB system, described in Schachner et al. (52). The AEB was calibrated to an individual system by using the following system parameters, which are in line with the suggestions of the Prospective Effectiveness Assessment for Road Safety (PEARS) consortium (53): Maximum sensor range in [m], Azimuth sensor opening angle [°], Azimuth sensor resolution [°], Trigger at which braking is induced – implemented based on Time to Collision (TTC) [s], Brake Delay [s] and Braking Gradient [m/s^3^]. The parameters of the virtual AEB system were selected as described in Schachner et al. (52). For each scenario, the following obtained quantities are stored in a CSV file: IDs, Collision speed baseline, Collision point baseline, Collision angle baseline, Collision speed VRU, Collision speed AEB, Collision point AEB, Collision angle AEB. The AEB system intervenes in many cases of an impending accident, which alters the collision scenario in comparison to the baseline. This section describes the calculation of new collision scenarios, which are determined by the following quantities: Collision angle A , Collision point CP , Vehicle collision speed ${\hat{v}}_{Veh}$ , VRU collision speed ${\hat{v}}_{VRU.}$

To calculate the probability of a certain collision scenario, the obtained quantities are clustered. Obtained collision angles are clustered in 30° steps. The collision speed of the pedestrian is clustered in 1 km/h steps to retain high resolution. The collision point relative to the vehicle front has been clustered in bins of 5%, starting at −50%. All parameters describing a collision scenario and related clusters are shown in Table 1.

**Table 1:** Clustered quantities describing the collision scenario.

	Range	Interval for clusters
Collision speed vehicle ${\hat{v}}_{Veh}$	0–120 [km/h]	5 [km/h]
Collision speed VRU ${\hat{v}}_{VRU}$	0–20 [km/h]	1 [km/h]
Collision angle A	0–360 [°]	30 [°]
Collision point CP	−50 [%]– +50 [%]	5 [%]

Equation 2 describes the calculation of the probability for a specific collision scenario $P(A\cap CP\cap {\hat{v}}_{Veh}\;\cap \;{\hat{v}}_{VRU})\;$ , using the probability of a certain virtual testing scenario as described in chapter 2.3.


(2) $$\begin{array}{l} P\left(A\cap CP\cap {\hat{v}}_{veh}\cap {\hat{v}}_{VRU}\right)=\\ \;\;\;\;\;\;\sum_{i}P(A\cap CP\cap {\hat{v}}_{veh}\cap {\hat{v}}_{VRU}\;|\;Scenario_{i})\;\;\cdot \;\;P\left(Scenario_{i}\right) \end{array}$$

These collision scenarios, which are not avoided by the AEB system, are further investigated in in-crash simulations.

#### 2.4.1. Demonstration example with VIRTUAL VRU-pre-crash-tool

Pre-crash simulations are performed with the VIRTUAL VRU-pre-crash-tool (20). The entire source code is publicly available on https://OpenVT.eu, including instructions on setting up the tool and how to generate and simulate a scenario catalog.

A conceptual AEB system, modeled on previous studies (12, 14, 19), was applied, and a geometric sensor with a range of 60 m and a field of view of 60° was positioned 0.25 m behind the most frontal point of the vehicle (2.2 m ahead of vehicle CoG). The VRU was detected when fully in view for 150 ms while braking was induced when the TTC was ≤1 s. The geometric sensor was modeled with an Azimuthal resolution of 0.5° and a time resolution of 10 ms. Following the brake delay, the deceleration was increased in line with the braking gradient until the maximum acceleration (depending on road friction) was reached.

### 2.5. In-crash simulation

To evaluate the injury risk of the unavoidable virtual testing scenarios, in-crash simulations with HBMs and a FE car model were performed. The HBM should thereby be in line with the specifications described in the Euro NCAP TB024 (54). The collision speed of the vehicle and the VRU, the collision angle of the VRU and the collision point of the in-crash simulation are part of the specific collision scenario. The definition of the collision angle and collision point of the VRU with respect to the vehicle front can be seen in the Supplementary material and in Schachner et al. (52).

The Design of Experiments (DoE) method is required to select a reasonable number of simulations suitable for the in-crash simulation. A trade-off must be found between the feasibility of running simulations (due to high computational power needed for HBM simulations) and the accuracy of predicted injury risks for the whole range of scenarios. Based on feedback from original equipment manufacturers (who should use this method to evaluate their vehicles), 50 cases were considered as an appropriate number of simulations for each load case (baseline and with AEB) and anthropometry. Whether the number of simulations is sufficient to guarantee good injury prediction was tested using different procedures which are presented in the results.

Through our simulation matrix design, we aimed to address both occurrence probability of the scenarios and good space filling. If high occurrence probability scenarios had only been selected, the study area would not have been covered sufficiently. Another requirement was that the DoE be repeatable and reproducible (each user should achieve the same simulation matrix when applying the method). Furthermore, the used algorithms must be available open source as the integrated assessment framework, including the DoE, has to be openly available. Eventually, the DoE and the whole workflow should be easily managed without much effort by users.

With these restrictions in mind, the MaxPro Criterion was selected. For further information on the MaxPro Criterion, see Joseph et al. (55) and Joseph et al. (56). This criterion is commonly used in deterministic computer experiments and is currently state of the art. The MaxPro criterion should facilitate finding space-filling designs that ensure good projections to subspaces of the factors. One further advantage of the MaxPro criterion is that a certain number of scenarios can be selected by the occurrence probability, consequently rendering the remaining scenarios – based on the first selection – selected to obtain good space filling.

As a starting point for the DoE, the scenarios which were not avoided by the generic AEB system were weighted according to their occurrence probability. The 50 simulation cases have been selected such that 60% of the cases (30 cases) are selected by MaxPro from the scenarios, representing the upper 50% quantile based on the occurrence probability of the clustered collision scenario. The other 40% were selected by MaxPro from all scenarios.

#### 2.5.1. Demonstration example with generic car

To demonstrate the workflow, in-crash simulations with a generic car as well as the VIVA+ 50F and 50 M models were performed. The details of these in-crash simulations are described below.

The open-source VIVA+ 50F and 50 M (version 0.3.2),^5^ which have been previously validated on component and full-scale levels (38, 41, 57), were used in this study. The two different anthropometries (50 M and 50F) differ in terms of outer geometry, bone geometry and mass distribution. Simulations were performed in LS-Dyna, version R12.

For the VRU impacts, a generic car front exterior (GVE) representing a Sedan shape was used for simulation. Revision 3 of the official CoHerent (58) Generic Vehicle models was used as baseline, and a deformable windshield was included. The stiffness of the GVE’s different parts was re-evaluated and compared with available data from literature regarding different stiffness levels of the current European fleet. The results of the impactor tests can be found in the Supplementary material. The standing VIVA+ models were positioned in a pedestrian stance in accordance with the specifications of the Euro NCAP TB024 (54).

### 2.6. Injury risk evaluation

The LS-Dyna output (binout) files were post-processed using the Python library Dynasaur. For injury risk evaluation, kinematic head injury assessment (Head Injury Criterion (HIC), DAMAGE), strain-based rib fracture assessment (risk of 3+ fractured ribs) and strain-based lower extremity fracture assessment (risk of proximal, femur shaft and tibia shaft fracture) have been calculated. More detailed documentation of the applied injury risk functions is listed in Table 2 and the Supplementary material.

**Table 2:** Injury risk curves and predictors used in the study.

	Based on	Sources
HIC	Resultant Head CoG accelerations filtered with CFC1000	(30)
DAMAGE	DAMAGE Implementation in Dynasaur using head rotation sensors implemented in VIVA+ definition files, filtered with CFC60	(59–61)
Risk of 3+ fractured ribs	Risk per rib determined based on maximum strain per rib. Combined to overall risk of 3+ fractured ribs using probabilistic method.	(62, 63)
Proximal Femur Fracture Risk	Risk based on MPS99 using risk curves calibrated for VIVA+ model	(38)
Femur Shaft Fracture Risk	Risk based on MPS99 using risk curves calibrated for VIVA+ model	(38)
Tibia Shaft Fracture Risk	Risk based on MPS99 using risk curves calibrated for VIVA+ model	See Supplementary material

A metamodel is used to also predict the injury probability of the unavoidable cases, which are not part of the in-crash simulation matrix.

After predicting the injury probability for the individual collision scenarios, the overall injury probability for each injury criterion can be calculated. The overall injury probability is calculated by adding the injury probability of each collision scenario multiplied by its occurrence probability.

#### 2.6.1. Demonstration example with specific metamodel

The development of the metamodel was done using the openly available python library Scikit-learn (64). With this library, it is possible to develop different metamodels based on a set of simulation results. The GaussianProcessRegressor (65–67) was used in these particular cases as it showed the best results, and the Matern kernel, with its default values, was also used. The other parameters of the GaussianProcessRegressor were determined with the help of the GridSearchCV algorithm of the Scikit-learn (64) package. This algorithm can find the optimized parameters with the help of a cross-validated grid-search over the parameter grid. A summary of the used values is shown in Table 3.

**Table 3:** Parameters of the GaussianProcessRegressor for the metamodel created with Scikit-learn (64).

Name	Value
kernel	Matern
n_restarts_optimizer	1,000
normalize_y	True
random_state	42

To analyze how well the metamodel is able to predict the overall injury risks and how many simulations are needed to create a sufficient working model, the root mean square error (RMSE) as well as the error of the overall injury prediction have been calculated for different numbers of VIVA+ 50F baseline simulations.

The RMSE is commonly used to evaluate the accuracy of a predictive model or the quality of predictions. RMSE measures the average magnitude of the differences between predicted values and the corresponding actual values. It is defined as shown in Equation 3

Equation 3 Definition of RMSE


(3) $$RMSE=\sqrt{\overset{n}{\underset{i=1}{\sum }}\frac{{\left(\hat{y_{i}}-y_{i}\right)}^{2}}{n}}$$

In this equation $\left(\hat{y_{i}}-y_{i}\right)$ represents the difference between the predicted and actual values for each data point. Squaring these differences emphasizes larger errors, and taking the mean calculates the average of these squared errors. Finally, taking the square root ensures that RMSE is in the same units as the original data, providing a measure of the average absolute error.

The error of the overall injury prediction is defined as the difference between the overall injury prediction observed in a ground truth and the overall injury prediction resulting from the metamodel created with different training and test sets. The ground truth in this study is based on 200 in-crash baseline VIVA+ 50F simulations selected by the DoE. The occurrence probability of the different collision scenarios was used as the weighting factor for calculating the overall injury risk.

To check whether 50 simulations are sufficient to create the metamodel the 200 the in-crash baseline VIVA+ 50F simulations have been used for verification purposes. The training set was increased in steps of ten simulations and it was checked how well the metamodel works. The rest of the simulations have been used to test the model (e.g., 10 Simulations for training and 190 simulations for testing) In the area to be investigated (50 Simulations), a finer sampling of one simulation was chosen in order to obtain a better overview.

## 3. Results

The introduced integrated assessment framework enables a holistic view on active and passive safety systems for VRU protection. With the help of the developed virtual testing scenarios that are based on real-world accident data and scaled on European level, and by means of pre-crash simulations, the performance of the active safety system can be evaluated. Together with the calculated occurrence probability for each virtual testing scenario, the total crash risk reduction can be calculated. For unavoided cases, the passive safety performance is assessed with the help of in-crash simulations. The change in collision parameters for the unavoided cases, by implementing an active safety system, also reduces the injury risk compared to the baseline. Detailed results and the process for evaluating the holistic assessment method are presented below.

### 3.1. Evaluation of the developed holistic assessment method

#### 3.1.1. In-depth accident data analyses – probability of accident parameters

The scale and shape parameters for the fitted Weibull distribution for the initial vehicle and pedestrian velocity can be seen in Supplementary Table S3 for the initial vehicle speed and Supplementary Table S4 for the initial pedestrian speed of the Supplementary material. By analyzing the data of three different European in-depth accident databases, we have created distributions for the motion sequence parameters. As the accident data was also collected according to the different injury severities a distinction could also be made between different injury severities. As for some conflict situations as well as for some injury severities no data was available in the accident databases (Supplementary Table S2) it was not possible to create the distributions for the initial speed for the whole population. In total, 531 reconstructed real-world accidents have been analyzed resulting in five different distributions for conflict situations resulting in slight injuries and each two different distributions for conflict situations resulting in severe and fatal injuries.

#### 3.1.2. Base level accident data analyses – weighting of conflict situations on European level

With the help of the base level accident database CARE the probabilities of the different conflict situations have been weighted on European level. Supplementary Table S5 shows the derived probabilities for each injury severity. It can be seen that for all clusters of conflict situations the injury severity minor holds the largest share between 74 and 66% followed by severe and fatal accidents. Supplementary Table S6 shows the derived probabilities for each type of conflict situation for the different injury severities. Within this analysis, it can be seen that crossing scenarios make up the largest proportion of all injury severities. By including also the road conditions dry and not-dry in the analyses it was also possible to distinguish between these parameter (Supplementary Table S7). The calculated probabilities are used for creating the virtual testing scenarios in a next step. The calculated weighting factors for different injury severities conflict situations and road conditions to be used for further analysis can also be found on the OpenVT platform (see footnote 3).

#### 3.1.3. Probability of a specific virtual testing scenario for pre-crash simulations

With the help of the probabilities for accident parameters and the weighting of the conflict situations on European level it was possible to retrieve the probability for a specific virtual testing scenario. The catalog of all created virtual testing scenarios can be found on the OpenVT platform (see footnote 2) together with the pre-crash tool used in this study. In total 61,914 virtual testing scenarios have been derived.

#### 3.1.4. Pre-crash simulations

**Figure 4:**
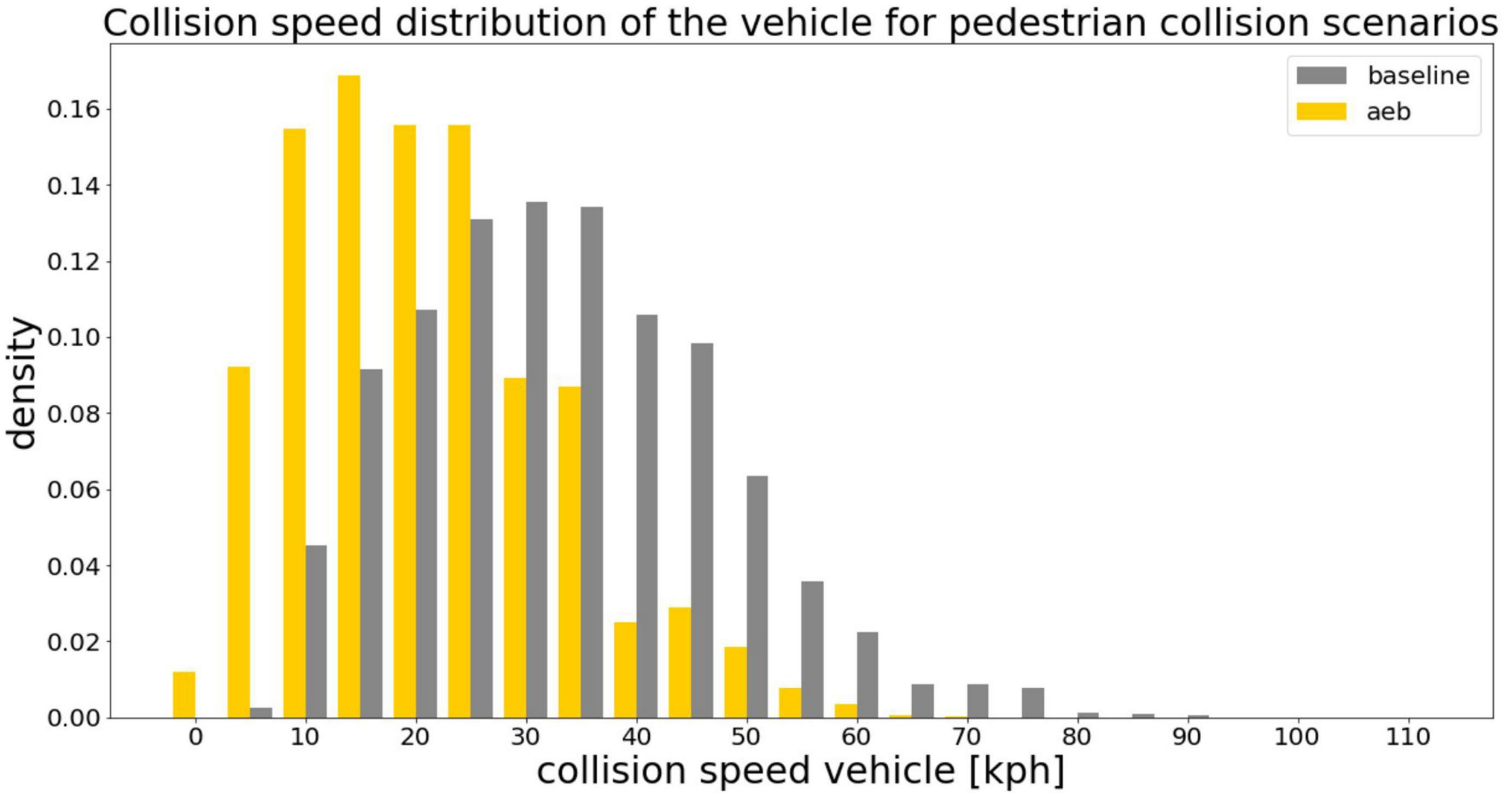
Results of the pre-crash simulations for car-pedestrian baseline (*n* = 61.914) and AEB (*n* = 37,833) cases. The step size of the histogram was chosen to be 5 km/h why there is also a small proportion of collision speeds from 0 to 5 km/h in the AEB cases. The avoidable cases are not shown in the histogram.

In total, 61,914 virtual testing scenarios have been derived from the different car-pedestrian cases. With the help of the implemented AEB system, 24,081 of those cases could have been avoided. Although 37,833 cases were unavoidable by the AEB system, considering the occurrence probability of the virtual testing scenarios, a total crash risk reduction of 81.70% was achieved. The results of the pre-crash simulations can be seen in Figure 4, where grey bars represent the baseline occurrence probabilities and yellow bars represent the probabilities based on the simulations with the AEB system. A clear trend toward lower collision speeds can be seen by implementing an AEB system. By clustering the baseline and unavoidable scenarios as described in 2.4 and calculating the related occurrence probability in total 3,953 baseline and 5,134 unavoidable in-crash scenarios remain for which the overall injury risk has to be predicted. The reason why there are more scenarios after implementing an AEB system is that there are more impact points and impact angles than in the baseline scenarios, but with considerably lower collision speeds.

#### 3.1.5. In-crash simulations and overall injury risk prediction for VIVA+ 50F baseline cases

Based on the MaxPro criterion with the help of the DoE (see also 2.5 for the DoE) 200 VIVA+ 50F baseline cases have been selected from the 3,953 baseline cases created in 3.1.4. These cases have then been taken into account for in-crash simulations and to determine the number of cases necessary for the metamodel.

**Figure 5:**
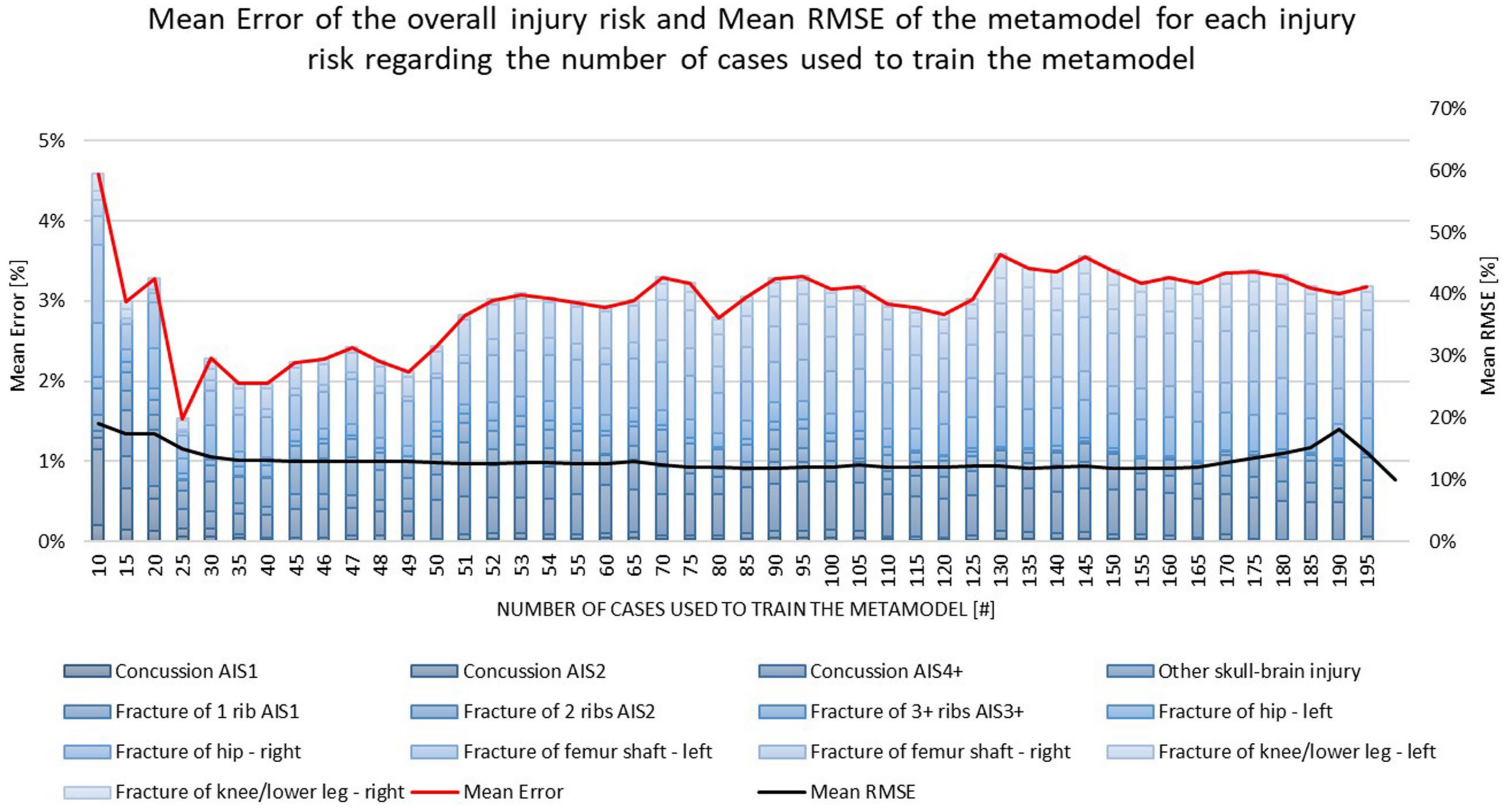
Mean Error of the overall injury risk and mean RMSE of the metamodel for each injury risk regarding the number of cases used to train the metamodel.

The result of the mean RMSE and the mean error of the overall injury prediction depending on the number of simulations used to create the metamodel can be seen in Figure 5. There, the mean values for both the RMSE and the error of the overall injury prediction are shown for all injury criteria. The values for each single criterion can be seen in the Supplementary material.

It is shown that when using only 10 Simulations to train the metamodel and 195 Simulations to test the developed model a mean RMSE of 19% and a mean error between the overall injury prediction and the ground truth (based on the 200 VIVA+ 50F simulations for verification) of 4.6% was observed. By using more simulations to train the metamodel the RMSE as well as the mean error decrease. The mean error reaches its minimum when using 25 simulations to train the metamodel. Although, when using 25 simulations to train the model the deviation between the overall injury prediction and the ground truth reaches a minimum the mean RMSE is still decreasing when adding more simulations to train the metamodel. In the range between 30 and 50 simulations, both the mean RMSE and the mean error seem to reach a kind of plateau. If more simulations are then added, the RMSE does not increase, but the mean error becomes considerably larger again from 50 simulations onwards and again reaches a kind of plateau at a higher level. With this in mind, it was chosen to use 50 simulations to train the metamodel for all use cases presented in this study.

### 3.2. Sex-specific holistic pedestrian protection evaluation

**Figure 6:**
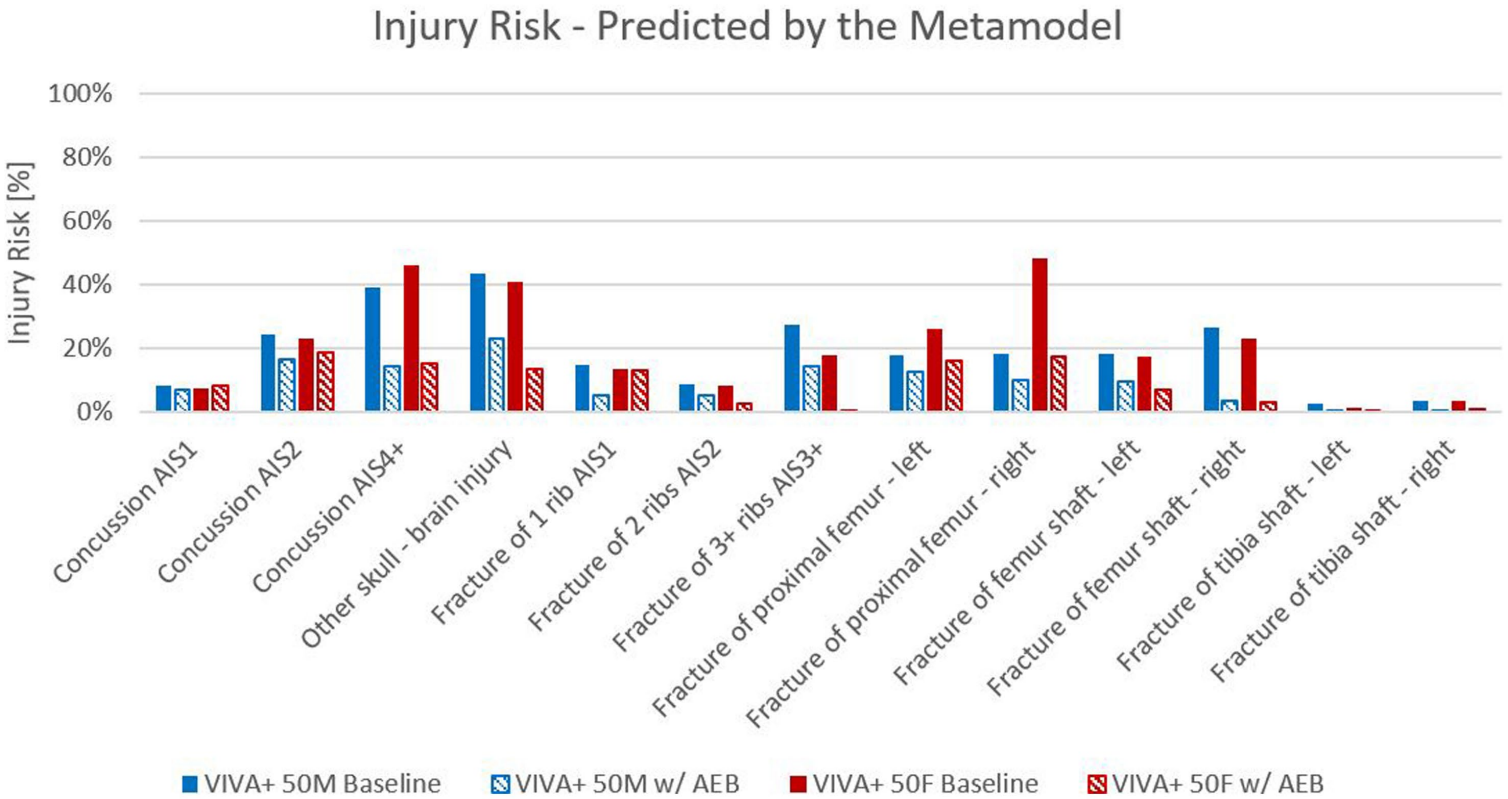
Comparison of VIVA+ 50F and 50 M simulations for baseline and AEB.

Looking at the injury risk for AIS4+ concussion injuries (Figure 6) revealed that, by implementing the generic AEB system, it was predicted that the injury risk would be reduced by approximately 20% for both males and females. A reduction was also observed regarding the other concussion injury levels. For skull injuries, a similar trend was observed, resulting in a lower injury risk due to the AEB system. For AIS3 + Rib injuries (3+ Ribs broken), the injury risk was also reduced. It was observed that females showed a lower AIS3+ rib injury risk than males for the baseline as well as for the AEB simulations. In contrast, for the right proximal femur fracture injury risk, females showed a higher fracture risk than males. The AEB system reduced the risk for both sexes. For femoral shaft fractures, a comparable injury risk was observed for males and females (for baseline and AEB simulations). The injury risk was also lowered by the AEB system for this type of injury. Only a very low injury risk was observed for tibia shaft fractures in both sexes, and this was further reduced by the AEB System. All the results are displayed in Figure 6.

## 4. Discussion

### 4.1. Limitations

The developed procedure is an important step toward holistic assessments and moves away from the exclusive use of standard-load case assessments for the prediction of real-world safety. However, the current study still underlies several limitations:

- For some categories of pedestrian conflict situations (e.g., LT/SDLD, RT/SDRD, …) no data was present within the investigated data sources. It remains unknown whether this is because they never occur or because these particular conflict situations have never been classified (which seems more likely).
- In the active safety assessment, currently, no interaction between the pedestrian and vehicle (i.e., avoiding reactions) is considered, and constant speeds have been chosen. Conversely, the considered AEB system is very generic and does not consider environmental conditions (e.g., fog), which limits the detection capabilities.
- For in-crash simulations, just two adult anthropometries and one age group have been considered. In the future, human variability should be further considered as well as VRU crashes with children. Furthermore, only one posture was considered for the pedestrian in our simulations. Analysis of real-world crashes has shown that avoidance-postures might be relevant (52) and should be considered in the future.
- Only injuries for which injury risk curves were available were assessed. By developing injury risk curves for other body regions in the future, even more injuries could be analyzed within this method.
- The generic Sedan model is simplified and provides a homogenous stiffness per structural part (i.e., the foam describing the structural behavior of the bonnet is homogenous, modeling a constant clearance throughout the whole bonnet). Therefore, there is also no difference in stiffness based on lateral position of the impact.

### 4.2. Evaluation of the developed holistic assessment method

#### 4.2.1. In-depth accident data analyses – probability of accident parameters virtual testing scenarios for pre-crash simulations

As already mentioned in the limitation certain categories of pedestrian conflict situations did not expose any cases in the investigated data sources. For this reason, it is important to have a more accurate accident classification in the future.

The Weibull distributions showed a better fit than Normal distributions for describing the initial velocities of vehicles and pedestrians. For cases involving low sample sizes, some deviations leading to poor *p*-values were observed in the Weibull distribution. Once the quality of data improves in the future, it would be possible to identify an even more appropriate distribution.

The classification of accident scenarios is not harmonized between European countries. The data available in CARE was therefore limited by the data sources, as was the assessment of injury severity. The overall probabilities have been calculated using the distribution of accidents to specific clusters for CARE applying the IPF method. The IPF method is useful when estimating probabilities based on databases that have been assumed to hold similar distributions of accidents. Please note that cluster probabilities might sometimes differ in CARE database. The CARE database consists of many more observations and might therefore provide more representative estimates of the cluster probabilities on European roads. Furthermore, for severe and fatal injuries in certain clusters, IPF results in uncertain estimates for conflict situations with few observations. Therefore, these values should be considered with caution, as they are associated with a relatively large degree of uncertainty. Exclusion of data belonging to certain clusters might change the results. This is particularly important for the cluster “*Unspecified*” and “*Others*” containing a large number of observations. As the probability of an “*unspecified*” type of conflict situation is not useful for our purposes (i.e., does not describe the design of the simulations and cannot be used for the specification of the virtual testing scenarios), these categories were excluded from the current analysis, although they can significantly affect the resulting probabilities. The plausibility checks with the in-depth databases showed that the derived probabilities are reasonable, at least for the countries where access to in-depth data is available.

Although accident data was not available for all conflict situations, it was possible through the stochastic determination of conflict situations and the IPF method to derive a catalog of virtual testing scenarios. Through this approach, 61,914 virtual testing scenarios have been derived. In contrast to test specifications described in UN Regulation No. 152 (68) and Euro NCAP (10) where only a few testing scenarios are defined this offers an approach to a more holistic view. Furthermore, it is possible to test different sensor settings of the AEB system through virtual testing. An accurate evaluation of the collision parameters (angle, point and velocity) needed for the in-crash simulation is also possible with this method.

#### 4.2.2. Pre-crash simulations

The amount of avoided accidents by means of pre-crash simulations was very high at 82.53%. Taking the before mentioned simplifications into account, the numbers seem reasonable. A mean collision speed reduction of the vehicle from 35.61 km/h to 23.69 km/h was accomplished by the generic AEB. This reduction of 33.5% is in line with the results of previous studies (12, 13), where an average collision velocity reduction of 33% was achieved through a similar setup of the AEB.

#### 4.2.3. In-crash simulations and overall injury risk prediction for VIVA+ 50F baseline cases

By analyzing the mean RMSE as well as the mean error between the overall injury prediction and the ground truth it was shown that 50 simulations are sufficient to predict the overall injury risk for different body regions. A maximum deviation between the ground truth and the overall injury prediction of 7% for right proximal femur fractures and a minimum deviation of 0.1% for brain injuries (AIS1) was observed. The mean deviation between the ground truth and the overall injury prediction for all injury criteria was 2.5% when using 50 Simulations to train the metamodel. Also the mean RMSE has reached a minimum value of 13% (absolute minimum 12%) with 50 simulations. The increase of the RMSE for some injury criteria (e.g., DAMAGE, HIC and fracture risk for 3+ ribs) after it has reached a minimum may be because of outliers (the data used for testing represents edge points as they are added last by the DoE space filling method) or overfitting of the model. These numbers show that it is possible to use 50 Simulations to predict the overall injury risk for the whole population of 3,953 in-crash scenarios.

**Figure 7:**
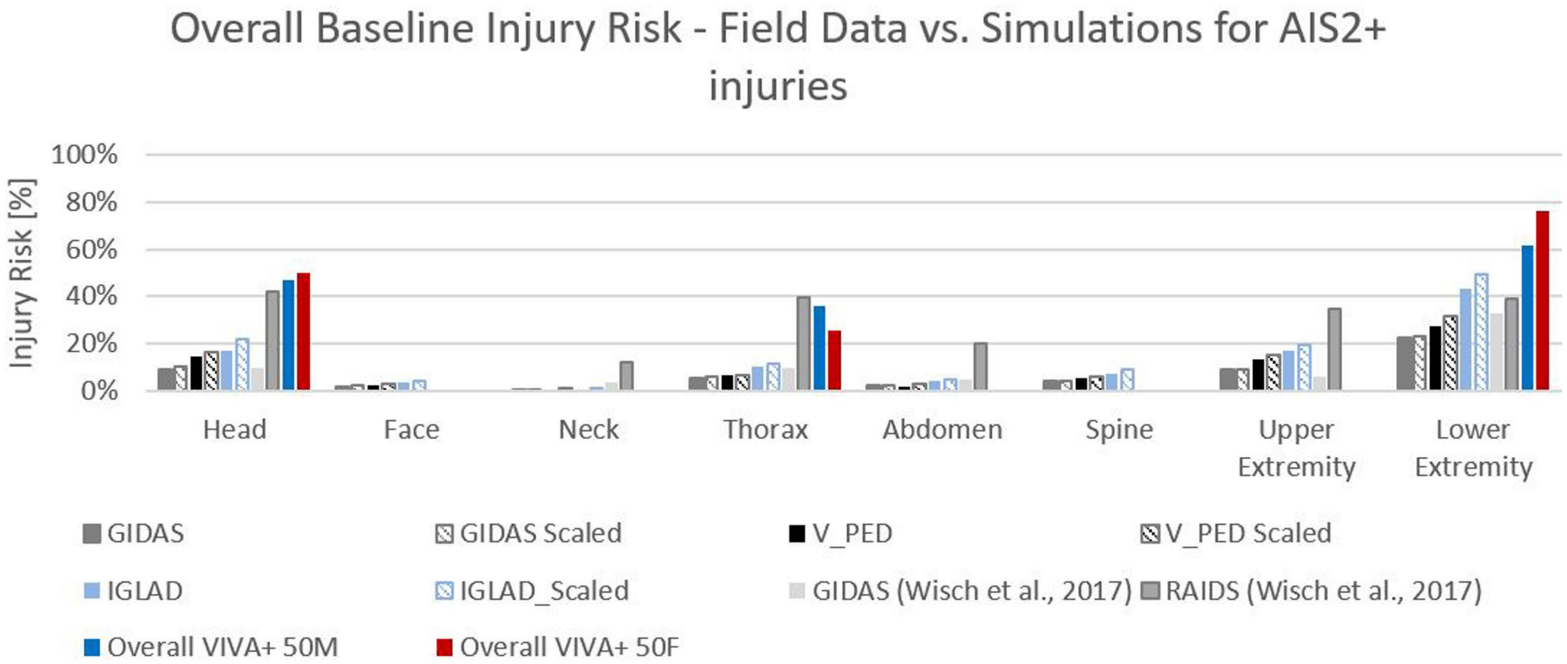
Comparison of different in-depth accident data with the overall baseline injury risk for VIVA+ 50F and 50 M for AIS2+ injuries.

To see how well the predicted injuries can be predicted, they have been compared with different field data. Therefore, the collected GIDAS and V_PED data was analyzed according to the injured body regions. The number of observable AIS2+ injuries in the collected data sample for each body region was analyzed. The data is presented, once unweighted (GIDAS) and once weighted, according to conflict situations probability (GIDAS Scaled) on European level derived from field data. Moreover, data from the Initiative of Global Harmonization of Accident Database (IGLAD) was analyzed and compared to the other data sets (69, 70). Also, data from the literature was used to compare our results with field data, namely data published by Wisch et al. (26). The comparison of field data with the overall injury risk for the VIVA+ 50F and 50 M can be seen in Figure 7. The different injuries per body region of the simulations have been summarized using the P_joint_ method proposed by the National Highway Traffic Safety Administration (71). It can be seen that the simulations are over predicting the injury risk compared to the data from the databases for most of the body regions and is therefore more on the conservative side. Only the RAIDS database prediction seems to work quite well. Similar trends, as in the databases, can be observed. Likewise, for the simulations, the lower extremities are the most frequently injured body region, followed by the head and the thorax when it comes to AIS2+ injuries. This might be caused by the design of the simulation matrix, where only one pose of the pedestrian was considered. Studies have shown (72, 73) that the used Euro NCAP (58) pose represents a kind of worst case for pedestrians involved in car collisions. By comparing the field data with the predicted injuries, it was seen that, when comparing the data to in-depth databases which include more serious and fatal injuries [e.g., RAIDS database (74)], the prediction is much more in line with the field data. Additionally, the distribution of injuries for the different body regions on AIS2+ level (lower extremities followed by the head and thorax) is in line with several studies (26, 32, 75, 76), with respect that upper extremity injuries have not been evaluated in this study.

Another limitation might be the used generic vehicle models, where the average stiffness and geometry does not necessarily lead to an average injury risk. A comparison to results where the method was applied with a state of the art (SotA) SUV shows that the overall results are comparable to a full FE vehicle model of a serial car.

### 4.3. Holistic sex-specific pedestrian protection

**Figure 8:**
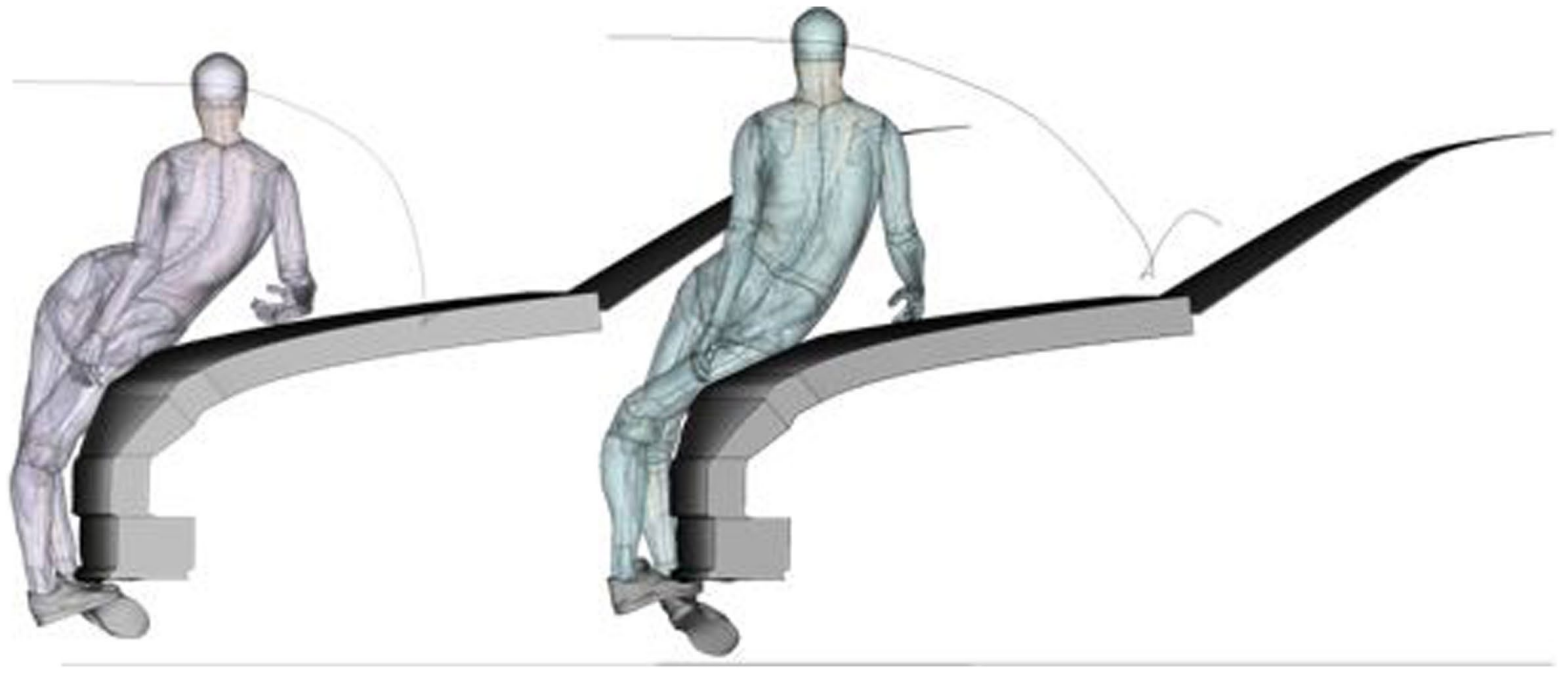
Pedestrian simulations with the VIVA+ 50F (left) and 50 M (right) model, impacted by the generic Sedan at the time of impact of the proximal femur on the bonnet leading edge for a perpendicular collision at 40 km/h. Additionally, the head trajectory and head impact location are visualized.

The differences in body shape, CoG and joint heights between the 50F and 50 M cause differences in the impact kinematics, impact locations of the different body parts and resulting injury risks as shown in Figure 8, where the impact location of the hip and head are compared between the two models. In accordance with field data studies (32), a higher proximal femur fracture risk was observed in the simulations with the 50F compared to the 50 M (48% vs. 18% overall risk in the baseline cases). While the femur head of the 50F model hits directly the BLE for an impact at 40 km/h, the femur head of the 50 M hits the bonnet at a later stage and more horizontal angle (Figure 8). The trend of higher risk of thoracic injuries for 50 M compared to 50F observed in real-world crashes (32) was also confirmed in the baseline simulations.

Moreover, as seen in the unavoidable cases, the injury risk for the average male and female are similar for brain injuries and femoral shaft fractures. In the baseline simulations, the 50 M has a 39% high risk of suffering an AIS4+ brain injury. After implementing an AEB system, the injury risk for AIS4+ brain injuries was reduced to 14%. Also for the 50F a similar trend was observed for AIS4+ brain injuries where the injury risk was lowered from 46 to 15% by the AEB system. With respect to the femoral shaft the injury risk was lowered from 22% to 12% for the 50 M and from 50% to 5% for the 50F. The average male has a higher risk of skull fractures (43% in baseline and 23% after AEB) and fractures of more than three ribs (28% in baseline and 15% after AEB) compared to the average female. The average female has a higher risk of proximal femoral fractures (37% in baseline and 17% after AEB) than the average male.

The presented method provides the opportunity to assess vehicle safety in a holistic way. The method can be used in the future to gain more knowledge regarding the injury risk of different road users involved in car accidents. Not only it is possible to look for countermeasures for a sex-equal protection for car fronts but also other anthropometries and children could be included in the future.

## 5. Conclusion

A novel methodology to assess VRU safety systems holistically was developed which allows for movement away from the exclusive use of standard-load case assessments. Safety assessment is performed over a wide range of scenarios while keeping the efforts required for simulations at a feasible level. As all tools used in the developed method are open-source available, it can be applied in the future by other researchers to more cars and scenario catalog.

In total, 61,914 virtual testing scenarios have been derived from the different car-pedestrian cases based on real-world crash scenario parameters. Considering the occurrence probability of the virtual testing scenarios, by implementing an AEB, a total crash risk reduction of 81.70% was achieved based on pre-crash simulations. It was shown that 50 in-crash simulations per load case are sufficient to create a metamodel for injury prediction, whereby the difference compared to 25 simulations was only max. 8% in terms of overall injury risk for the proximal femur fractures. For the in-crash simulations with the generic vehicle, it was also shown that the injury risk can be reduced by implementing an AEB, as compared to the baseline scenarios. The highest reduction can be observed for AIS4+ brain injuries where the injury risk was lowered from 39 to 14% for the 50 M and 46 to 15% for the 50F. Besides concussion injuries the 50 M showed the highest reduction in injury risk for femur shaft fractures where the risk was reduced from 26 to 3%. For 50F the injury risk for proximal femur fractures could be lowered remarkably from 48 to 17% by implementing an AEB system beside AIS4+ brain injuries. Current sex-related differences observed in the literature (50F has higher risk for proximal femur fractures and 50 M has higher risk for rib fractures) were also identified in the baseline simulations. This trend is still seen in our simulations after implementing an AEB system. The 50 M has still a higher fracture risk for more than three rips (14% compared to 0.5% of the 50F) and the 50F has still a higher risk for proximal femur fractures (17% compared to 11% of the 50 M).

With the developed method, the overall benefit of integrated VRU protection can be derived.

## Data availability statement

The data analyzed in this study is subject to the following licenses/restrictions: Raw data from accident databases from Austria, Germany, and Sweden are not publicly available (due to data protection regulations). Requests to access these datasets should be directed to CL, christoph.leo@tugraz.at and CK, corina.klug@tugraz.at. All necessary tools for the holistic VRU safety assessment are openly available on https://openvt.eu.

## Author contributions

CL carried out the study design, data analysis, simulations, and manuscript preparation. CK has designed the study and supervised the data analysis. MS has run the pre-crash simulations and developed the applied pre-crash tool. AF, EG, AL, MS, and FT have provided comments, feedback, and edited the manuscript. All the authors have read and approved the final manuscript.

## Funding

Open access funding was provided by Graz University of Technology Open Access Publishing Fund. This study has received funding from the European Union Horizon 2020 Research and Innovation Programme under Grant Agreement No. 768960.

## Conflict of interest

AF and FT were employed by Volvo Car Corporation.

The remaining authors declare that the research was conducted in the absence of any commercial or financial relationships that could be construed as a potential conflict of interest.

The reviewer RR declared a past co-authorship with the author CK to the handling editor.

## Publisher’s note

All claims expressed in this article are solely those of the authors and do not necessarily represent those of their affiliated organizations, or those of the publisher, the editors and the reviewers. Any product that may be evaluated in this article, or claim that may be made by its manufacturer, is not guaranteed or endorsed by the publisher.

## Footnotes

^1^ https://openvt.eu/visafe-vru/VISAFE-VRU/-/tree/master/Auxilaries/pre_crash_simulation

^2^ https://openvt.eu/virtual_precrash/vru-precrash-tool

^3^ https://openvt.eu/visafe-vru/VISAFE-VRU

^5^ https://vivaplus.readthedocs.io/en/latest/
