# Supplementary material for: Holistic pedestrian safety assessment for average males and females
Source: Front Public Health. 2023 Aug 17;11:1199949. doi: 10.3389/fpubh.2023.1199949 (PMC10476492; doi:10.3389/fpubh.2023.1199949)
Supplement: Supplementary file 1 [file Data_Sheet_1.pdf]

Supplementary Material

Table S-1 List of accident parameters which have been analyzed in the in-depth accident databases.

Parameter	from	to	resolution	Unit / parameters
Accident Location	-	-	-	Motorway, rural, urban
Age VRU	0	100	1	[years]
BMI VRU	0	50	1	[kg/m^2]
Braking Deceleration	0	1	0.01	[g]
Collision Speed Vehicle t0	0	100	1	[km/h]
Collision Speed VRU t0	0	100	1	[km/h]
Daytime	00:00	24:00	00:15	[h:min]
Sex VRU	-	-	-	Male, female
Height VRU	0	200	1	[cm]
Injured Body Regions VRU	1	9	1	Head, face, neck, thorax, abdomen, spine, upper extremity, lower extremity, unspecified
Road Conditions	-	-	-	Dry, glazed frost, slippery, wet, other
Visual Obstructions	-	-	-	Non, static, dynamic
Initial Speed Vehicle(v0)	0	100	1	[km/h]
Initial Speed VRU(v0)	0	100	1	[km/h]
Weather Conditions	-	-	-	Bright, cloudy, rain, storm, snow, fog, other
Weight VRU	0	200	1	[kg]
Year	2000	2018	1	[year]
Light Conditions	-	-	-	Darkness, dawn/twilight, daylight, artificial light
Deceleration Distance	0	100	1	[m]
Road Situation	-	-	-	Footpath, cycling/rambling path, other
Speed Limits	0	150	1	[km/h]

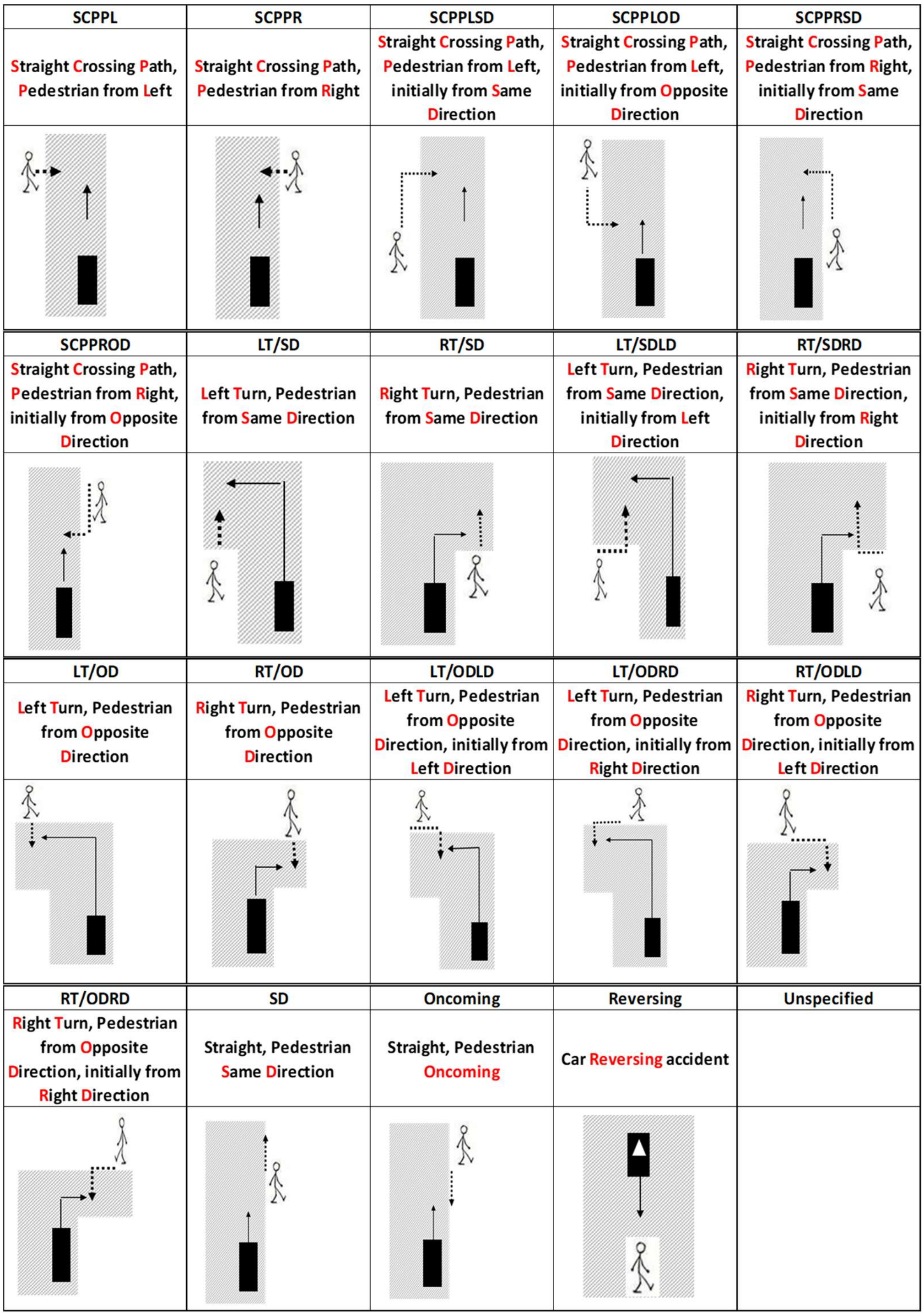

Figure S-1 Conflict situations for vehicle - pedestrians.

Table S-2 Number of accidents from V_PAD, GIDAS and CEDATU according to the injury severity and conflict situations.

Pedestrian Conflict Situations	Minor				Severe				Fatal			
	V_PAD	GIDAS	CEDATU	∑	V_PAD	GIDAS	CEDATU	∑	V_PAD	GIDAS	CEDATU	∑
SCPPL	15	94	6	115	2	13	18	33	-	3	76	79
SCPPR	25	163	6	194	1	13	16	30	-	1	37	38
SCPPLSD	-	-	-	-	-	-	2	2	-	-	1	1
SCPPLOD	-	-	-	-	-	-	-	-	-	-	-	-
SCPPRSD	2	-	-	2	-	-	-	-	-	-	5	5
SCPPROD	-	-	-	-	-	-	2	2	-	-	-	-
details unknown	-	-	-	-	-	-	-	-	-	-	-	-
LT/SD	-	15	-	15	-	-	2	2	-	-	-	-
RT/SD	-	8	1	9	-	-	1	1	-	-	-	-
LT/SDLD	-	-	-	-	-	-	-	-	-	-	-	-
RT/SDRD	-	-	-	-	-	-	-	-	-	-	-	-
details unknown	-	-	-	-	-	-	-	-	-	-	-	-
LT/OD	-	16	-	16	-	-	-	-	-	-	1	1
RT/OD	-	6	-	6	-	1	-	1	-	-	-	-
LT/ODLD	-	-	-	-			1	1	-	-	1	1
LT/ODRD	-	-	-	-	-	-	-	-	-	-	-	-
RT/ODLD	-	-	-	-	-	-	-	-	-	-	-	-
RT/ODRD	-	-	-	-	-	-	-	-	-	-	-	-
details unknown	-	-	-	-	-	-	-	-	-	-	-	-
SD	-	4	-	4	-	1	1	2	-	-	19	19
Oncoming	-	1	-	1	-	2	-	2	-	-	10	10
details unknown	-	-	-	-	-	-	-	-	-	-	-	-
Reversing	-	23	1	24	-	-	3	3	-	-	3	3
Stationary Pedestrian	-	4	-	4	-	-	1	1	-	-	-	-
Unspecified	1	20	-	21	1	-	-	1	1	-	-	1
				411				81				158

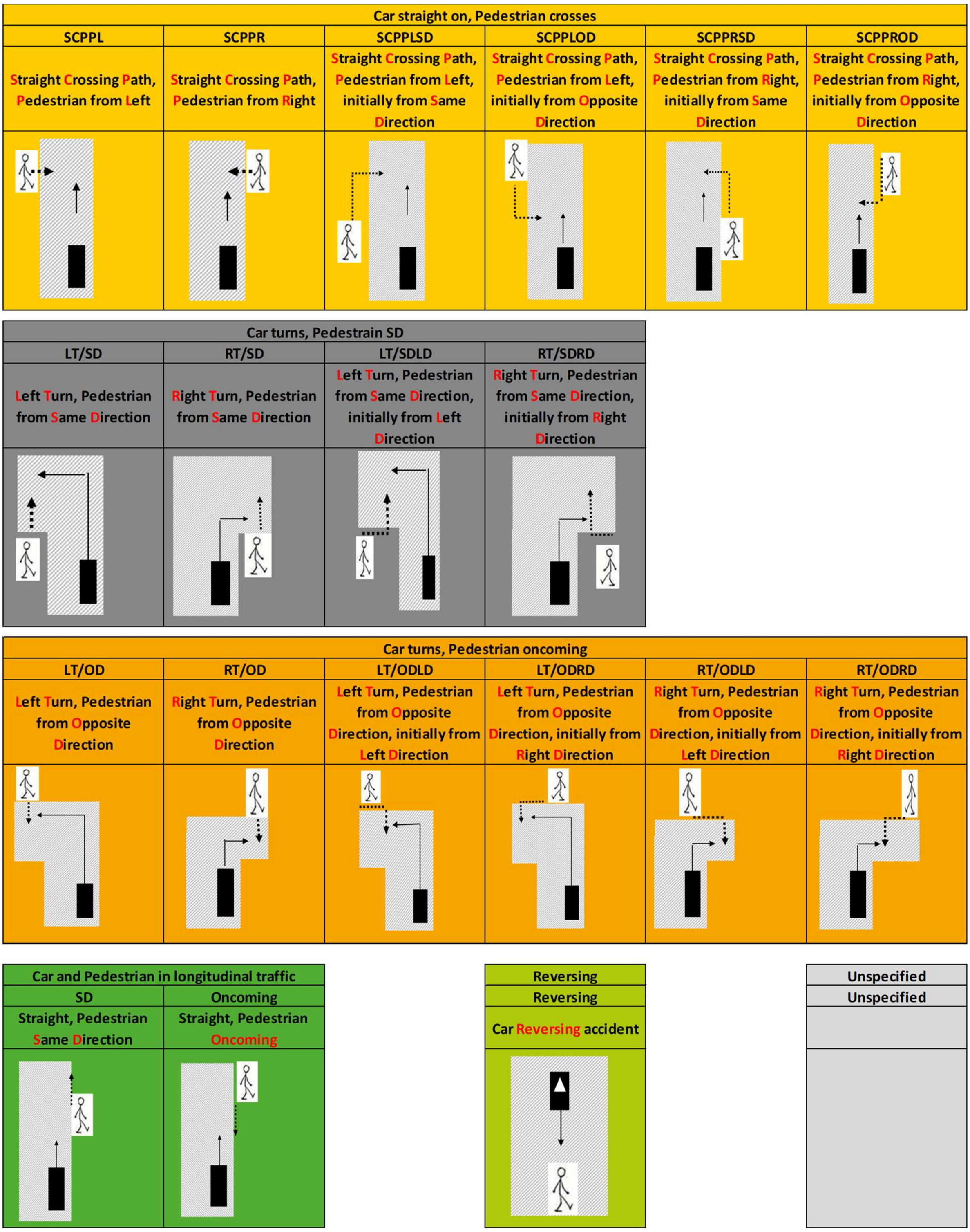

Figure S-2 Clustering of conflict situations for vehicle - pedestrians.

Table S-3 Parameters of fitted Weibull distribution to accident sample data for different detailed conflict situations and different injury severities for the initial vehicle speed in pedestrian accidents.

Situation	Severity	Number of Cases:	Shape	Scale	P-value
SCPPL	slight	112	2.64	38.81	0.004
SCPPR	slight	183	2.78	36.49	0.00
LT/SD	slight	15	1.94	20.71	0.148
LT/OD	slight	14	3.16	22.33	0.602
SD	slight	15	1.94	20.71	0.168
Reversing	slight	16	3.07	8.39	0.044
Unspecified	slight	18	2.28	33.66	0.532
Overall	slight	382	2.27	34.43	0.00
SCPPL	severe	32	2.99	54.14	0.098
SCPPR	severe	28	2.89	43.50	0.178
Overall	severe	73	2.48	48.70	0.032
SCPPL	fatal	67	3.51	64.09	0.144
SCPPR	fatal	31	2.87	62.95	0.422
Overall	fatal	127	3.32	66.70	0.160

Table S-4 Parameters of fitted Weibull distribution to accident sample data for different detailed conflict situations and different injury severities for the initial pedestrian speed in pedestrian accidents.

Situation	Severity	Number of Cases:	shape	scale	p-value
SCPPL	slight	112	2.02	7.61	0
SCPPR	slight	183	1.82	7.11	0
LT/SD	slight	15	2.23	6.93	0.306
LT/OD	slight	14	1.91	6.20	0.278
SD	slight	15	2.23	6.93	0.294
Reversing	slight	16	1.94	4.97	0.048
Unspecified	slight	18	1.45	6.97	0.016
Overall	slight	382	1.87	7.01	0
SCPPL	severe	32	1.90	7.48	0.136
SCPPR	severe	28	2.00	7.04	0.888
Overall	severe	73	1.95	7.06	0.038
SCPPL	fatal	67	2.16	6.43	0.002
SCPPR	fatal	31	2.41	5.92	0.938
Overall	fatal	127	2.14	5.81	0

Table S-5: Injury severity per conflict situation cluster

Cluster		Minor	Severe	Fatal	Total
Car straight on, Pedestrian crosses		71.03%	26.77%	2.20%	100.00%
Car turns, Pedestrian SD +	Car turns, Pedestrian oncoming	73.78%	18.65%	7.58%	100.00%
Car and Pedestrian in longitudinal traffic		66.07%	25.59%	8.34%	100.00%

Table S-6: Occurrence probabilities at minor/severe/fatal severity

Cluster	Conflict situation	Minor	Severe	Fatal
Car straight on, Pedestrian crosses	SCPPL	32.3%	42.9%	53.7%
	SCPPR	53.2%	41.9%	24.9%
	SCPPLSD	0.1%	2.0%	0.7%
	SCPPLOD	0.0%	0.0%	0.0%
	SCPPRSD	0.3%	0.0%	3.3%
	SCPPROD	0.0%	2.0%	0.0%
	Car straight on, Pedestrian crosses	**85.8%**	**88.9%**	**82.5%**
Car turns, Pedestrian SD	LT/SD	3.9%	4.2%	0.0%
	RT/SD	1.9%	1.0%	0.0%
	LT/SDLD	0.0%	0.0%	0.0%
	RT/SDRD	0.0%	0.0%	0.0%
	Car turns, Pedestrian SD	**5.8%**	**5.2%**	**0.0%**
Car turns, Pedestrian oncoming	LT/OD	3.6%	0.0%	1.7%
	RT/OD	1.2%	1.0%	0.0%
	LT/ODLD	0.0%	1.0%	1.7%
	LT/ODRD	0.0%	0.0%	0.0%
	RT/ODLD	0.0%	0.0%	0.0%
	RT/ODRD	0.0%	0.0%	0.0%
	Car turns, Pedestrian oncoming	**4.8%**	**2.1%**	**3.3%**
Car and Pedestrian in longitudinal traffic	SD	2.4%	2.2%	9.4%
	Oncoming	1.2%	1.6%	4.7%
	Car and Pedestrian in longitudinal traffic	**3.6%**	**3.8%**	**14.1%**
		100.0%	100.0%	100.0%

Table S-7: Road conditions at minor/severe/fatal severity

Cluster	Conflict situation	Minor		Severe		Fatal	
		dry	non-dry	dry	non-dry	dry	non-dry
Car straight on, Pedestrian crosses	SCPPL	61.0%	39.0%	74%	26%	55%	45%
	SCPPR	71.0%	29.0%	59%	41%	69%	31%
	SCPPLSD	100.0%	0.0%	100%	0%	100%	0%
	SCPPLOD	0.0%	0.0%	0%	0%	0%	0%
	SCPPRSD	0.0%	100.0%	100%	0%	100%	0%
	SCPPROD	0.0%	0.0%	0%	100%	0%	0%
Car turns, Pedestrian SD	LT/SD	35.0%	65.0%	25%	75%	0%	0%
	RT/SD	46.0%	54.0%	0%	100%	0%	0%
	LT/SDLD	0.0%	0.0%	0%	0%	0%	0%
	RT/SDRD	0.0%	0.0%	0%	0%	0%	0%
Car turns, Pedestrian oncoming	LT/OD	66.0%	34.0%	0%	0%	0%	100%
	RT/OD	50.0%	50.0%	100%	0%	0%	0%
	LT/ODLD	0.0%	0.0%	0%	100%	100%	0%
	LT/ODRD	0.0%	0.0%	0%	0%	0%	0%
	RT/ODLD	0.0%	0.0%	0%	0%	0%	0%
	RT/ODRD	0.0%	0.0%	0%	0%	0%	0%
Car and Pedestrian in longitudinal traffic	SD	67.0%	33.0%	0%	80%	75%	25%
	Oncoming	64.0%	36.0%	33%	67%	67%	33%

In-Crash Simulation definition

The impact point between the HBM and the car is defined as shown in Figure S-3. This means that for an impact point of 0%, the origin of the global HBM coordinate system is in line with the center of the car front. -50% means that the origin of the global HBM coordinate system is at the outer most left of the car front and +50% means that the origin of the global HBM coordinate system is at the outer most right of the car front. The car front width is thereby defined as the distance between the exterior points of the bonnet leading edge.

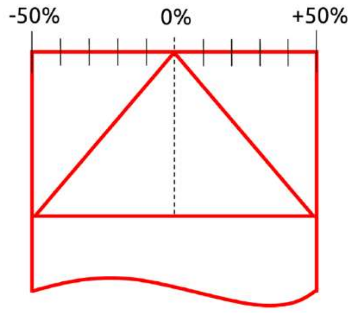

Figure S-3 Impact Point

The impact angle between the HBM and the car is defined as shown in Table S-8. This means that for the 0° position, the axes of the HBM coordinate system and the vehicle system are all pointing in the same direction. For the 90° position, the x-axis of the HBM coordinate system is pointing in positive y-axis direction of the vehicle coordinate system. For the 180° position, the x-axis of the HBM is pointing in negative x direction of the vehicle coordinate system. For the 270° position, the x-axis of the HBM is pointing in negative y-axis direction of the vehicle coordinate system.

Table S-8 Impact Angle (with coordinate system: x positive is pointing forward in vehicle direction, y positive is pointing to the left and z-positive is pointing upwards)

0° impact angle and 0% impact point	90° impact angle and 0% impact point	180° impact angle and 0% impact point	270° impact angle and 0% impact point
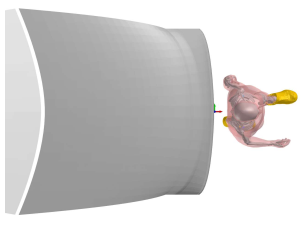	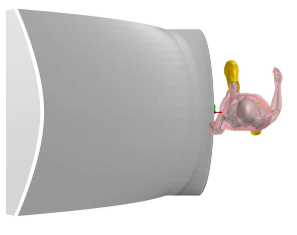	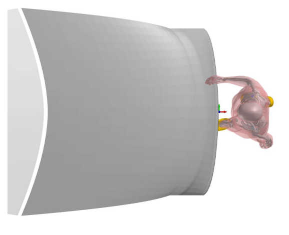	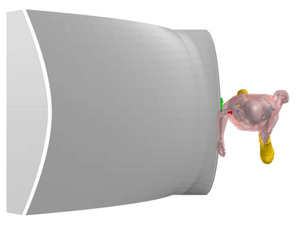

The HBMs should be positioned 1mm above the ground. Gravity should be used for all simulations.

GVE Validation

Supplementary Material

The stiffness characteristics of the spoiler, bumper and bonnet leading edge of the GVE models were compared to corridors from current fleet data from Feist et al. (2019). The results of the impactor tests can be seen in Table S-9.

Table S-9 Response of the impactor test (black) compared to the results reported in Feist et al. (2019)

Oblique spoiler test (SPV)	Bumper impact, horizontal (BMP)	Bonnet leading edge impact, oblique (BLE)
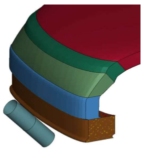	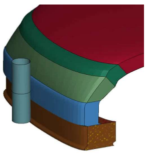	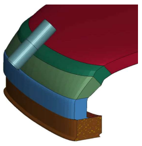
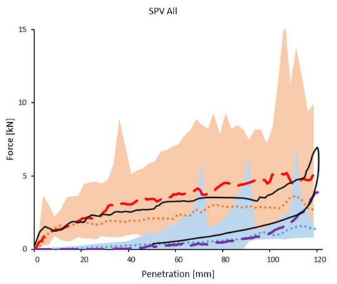	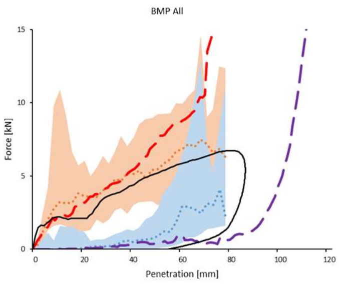	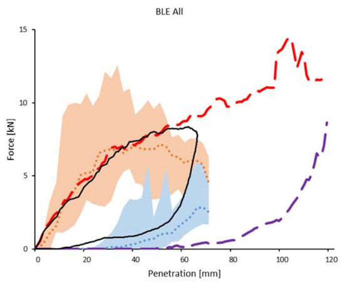
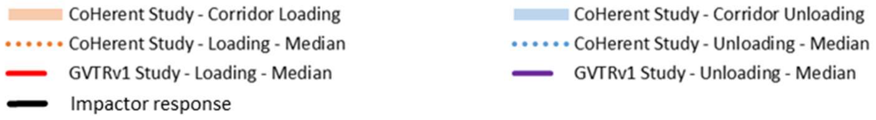		

As in the original GV models, according to Euro NCAP Technical Bulletin TB024, the windscreen was modeled rigid for injury assessment; this must be adjusted. Therefore, the windscreen was modeled with a solid PVC layer coffered by two shell layers of glass. With the help of a head impactor test on the outside of the windscreen, the response of the windscreen was compared with the values reported in Alvarez and Kleiven (2016). Therefore, the impact velocity of the 4.58kg head impactor was adjusted to 10.24m/s to reach the same kinetic energy of 240J as reported by Alvarez and Kleiven (2016).

Injury risk curves and predictors used in this study

$$P = \phi\left(\frac{ln(HIC15) - 6.96352}{0.84664}\right)$$

Equation S-1 Skull Fracture Risk based on Head Injury Criterion (HIC) (Hertz, 1993)

$$P = 1 - e^{\left(e^{\left(\frac{1}{b}*\ln(d*x+c)-\frac{a}{b}\right)}\right)}$$

Equation S-2 Brain Injury Risk Function for a Concussion (JAMA, 2019)

$$Fracture\ risk\ (strain, AGE) = \frac{1}{2} + \frac{1}{2}\mathrm{erf}\left[\frac{\ln(strain) - (\beta_0 + \beta_1 * AGE)}{\sqrt{2} * \alpha}\right]$$

with: $\alpha = 0.3026$

$\beta_0 = -2.9866$

$\beta_1 = -0.0130$

Equation S-3 Rib fracture risk based on max. principal strain and age (Larsson et al., 2021)

$$P = 1 - e^{-\left(\frac{x}{\lambda}\right)^{\mathrm{k}}} \qquad for\ x \geq 0$$

with: $shape\ k = 2.22438$

$scale\ \lambda = 0.01492$

Equation S-4 Injury Risk for a Femur Proximal Fracture (Schubert et al., 2021)

$$P = 1 - e^{-\left(\frac{x}{\lambda}\right)^{\mathrm{k}}} \qquad for\ x \geq 0$$

with:

$$shape\ k = 2.74258$$

$$scale\ \lambda = 0.034152$$

Equation S-5 Injury Risk for a Femur Shaft Fracture (Schubert et al., 2021)

$$P = 1 - e^{-\left(\frac{x}{\lambda}\right)^{\mathrm{k}}} \qquad for\ x \geq 0$$

with:

$$shape\ k = 5.04817$$

$$scale\ \lambda = 0.011885$$

Equation S-6 Injury Risk for a Tibia Shaft Fracture

The injury risk curve for the tibia shaft has been established using a method corresponding to that employed for the femur bone by conducting the needed tests, followed by a detailed analysis of the test results. Findings of this study, along with the official research paper, are currently pending release.

RMSE an overall injury prediction depending on the number of simulations used to train the metamodel for each injury criteria

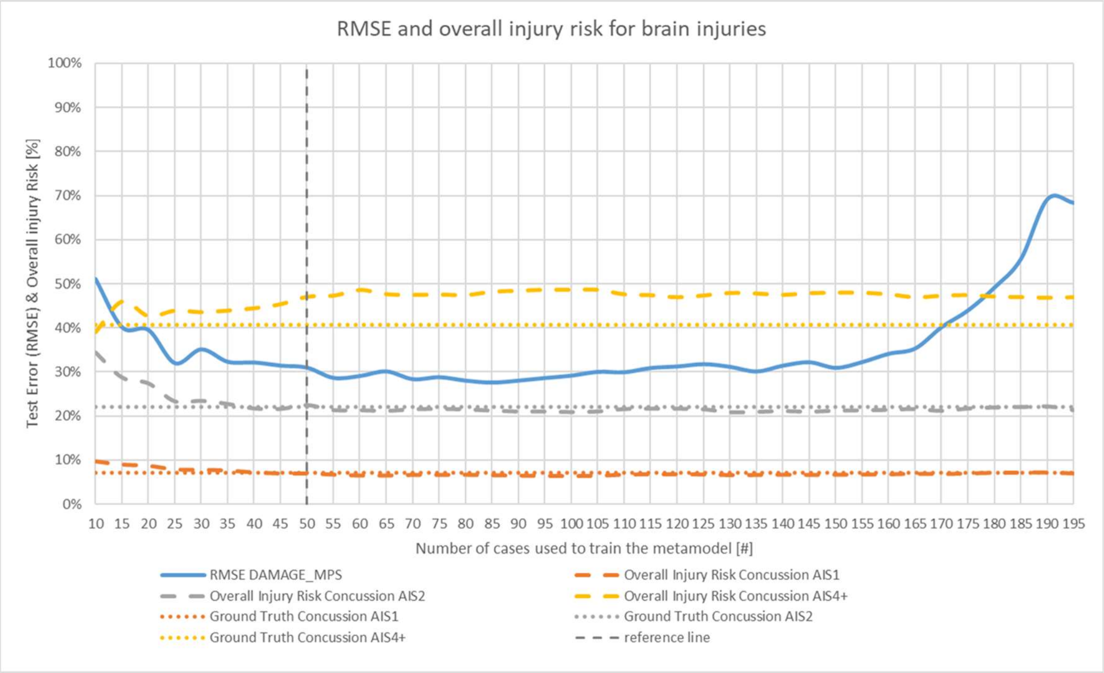

Figure S-4 RMSE and overall injury risk for brain injuries

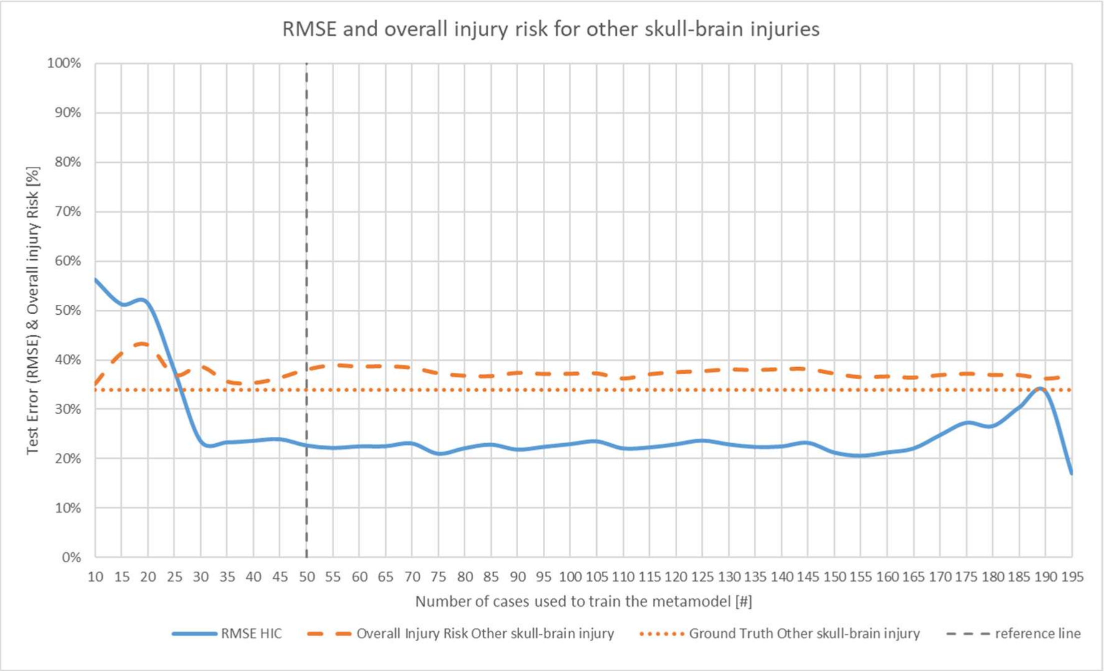

Figure S-5 RMSE and overall injury risk for other skull-brain injuries

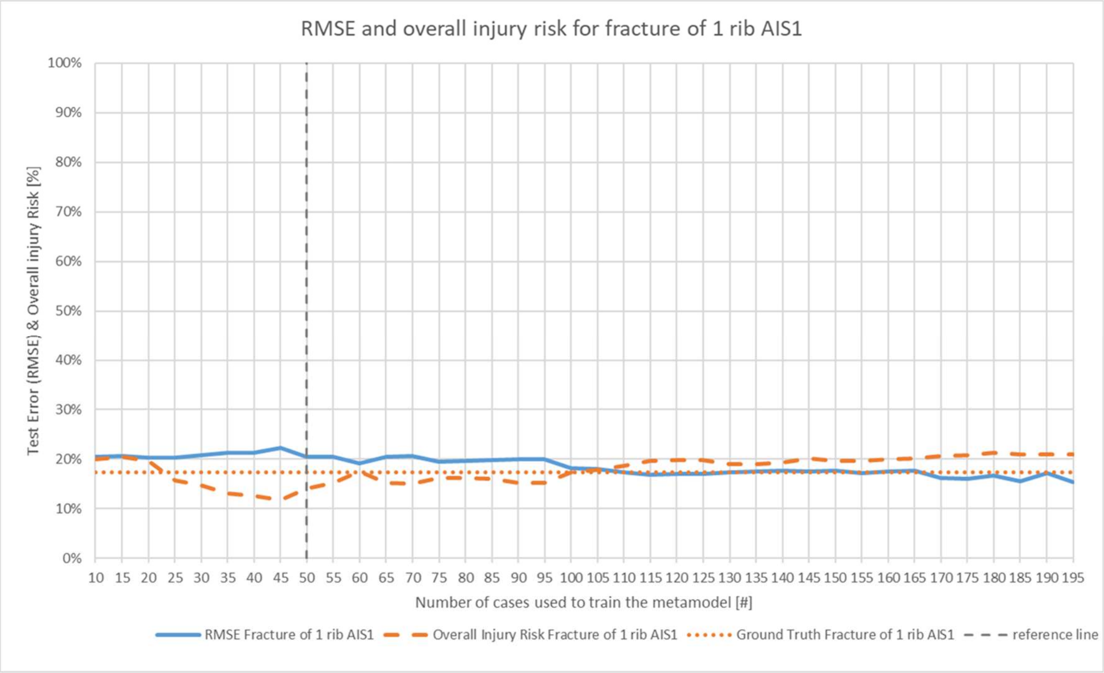

Figure S-6 RMSE and overall injury risk for fracture of 1 rib AIS1

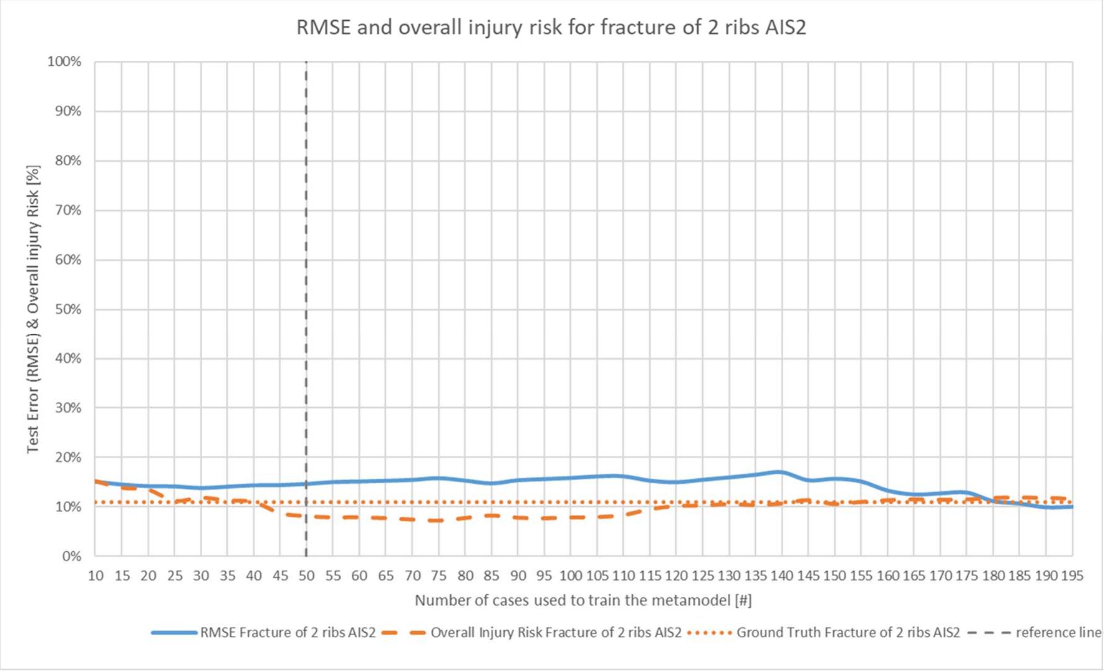

Figure S-7 RMSE and overall injury risk for fracture of 2 ribs AIS2

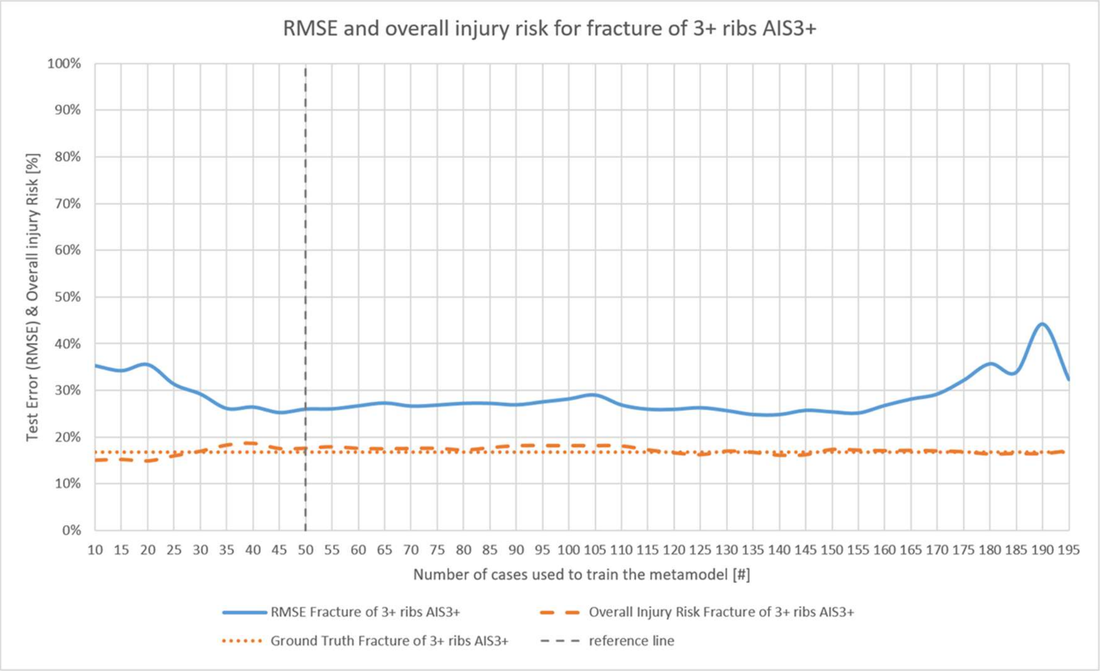

Figure S-8 RMSE and overall injury risk for fracture of 3+ ribs AIS3+

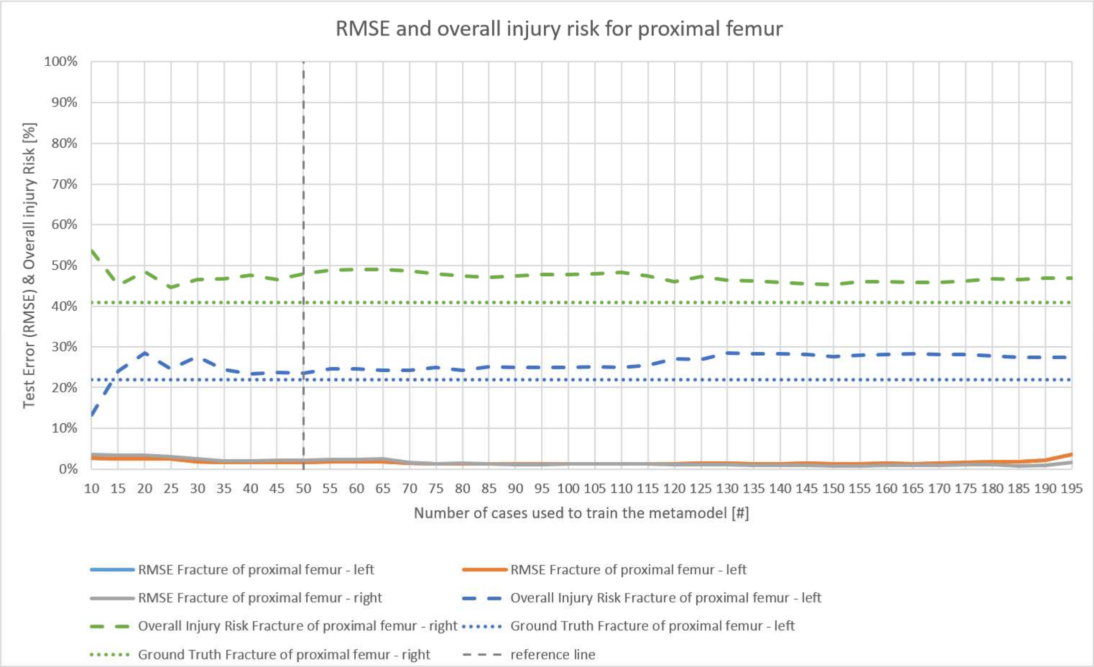

Figure S-9 RMSE and overall injury risk for proximal femur

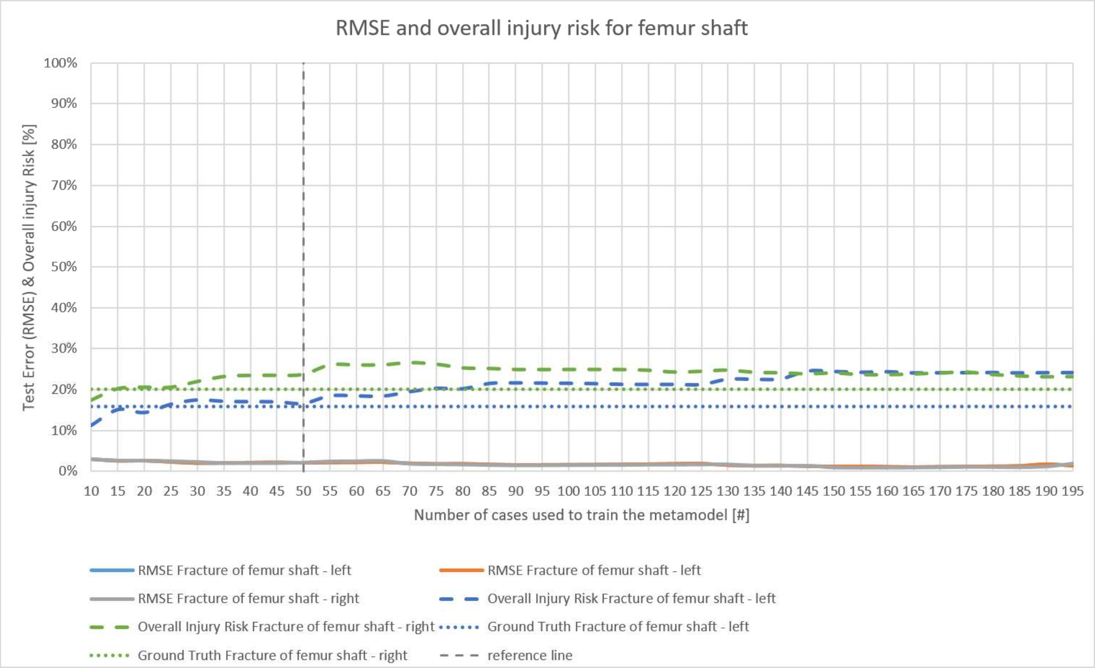

Figure S-10 RMSE and overall injury risk for femur shaft

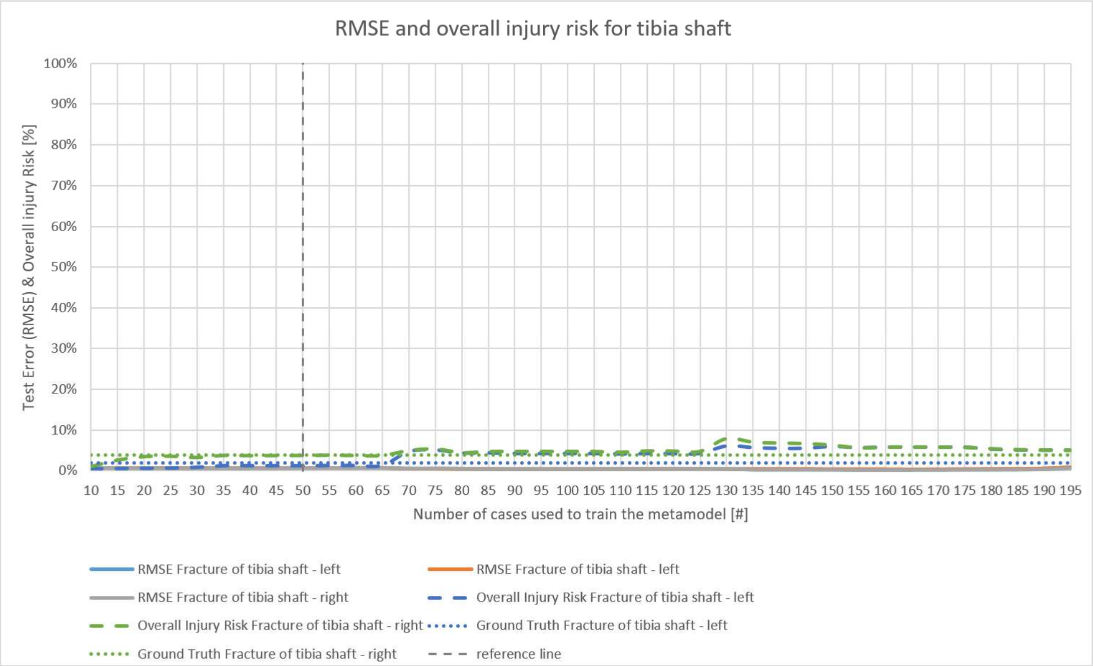

Figure S-11 RMSE and overall injury risk for tibia shaft

A. REFERENCES

Alvarez, V. S., and Kleiven, S. (2016). "Importance of Windscreen Modelling Approach for Head Injury Prediction," in *2016 IRCOBI Conference Proceedings*, ed. International Research Council on the Biomechanics of Injury (IRCOBI), 813–830.

Feist, F., Sharma, N., Klug, C., Roth, F., Schinke, S., Besch, A., et al. (2019). "GVTR: A Generic Vehicle Test Rig Representative of the Contemporary European Vehicle Fleet," in *The 26th ESV Conference Proceedings*, ed. NHTSA.

Hertz, E. (1993). "A note on the head injury criterion (HIC) as a predictor of the risk of skull fracture," in *37th Annual Conference: American Association for Automotive Medicine (AAAM)*, 303–312.

JAMA (2019). "Development of a brain injury criterion and associated injury risk curves relevant for car crash safety evaluation,", November 5.

Larsson, K.-J., Blennow, A., Iraeus, J., Pipkorn, B., and Lubbe, N. (2021). Rib Cortical Bone Fracture Risk as a Function of Age and Rib Strain: Updated Injury Prediction Using Finite Element Human Body Models. *Front Bioeng Biotechnol* 9, 677768. doi: 10.3389/fbioe.2021.677768

Schubert, A., Erlinger, N., Leo, C., Iraeus, J., John, J., and Klug, C. (2021). "Development of a 50th Percentile Female Femur Model," in *2021 IRCOBI Conference Proceedings*, ed. International Research Council on the Biomechanics of Injury (IRCOBI), 308-332.
